# Curing Cats with Feline Infectious Peritonitis with an Oral Multi-Component Drug Containing GS-441524

**DOI:** 10.3390/v13112228

**Published:** 2021-11-05

**Authors:** Daniela Krentz, Katharina Zenger, Martin Alberer, Sandra Felten, Michèle Bergmann, Roswitha Dorsch, Kaspar Matiasek, Laura Kolberg, Regina Hofmann-Lehmann, Marina L. Meli, Andrea M. Spiri, Jeannie Horak, Saskia Weber, Cora M. Holicki, Martin H. Groschup, Yury Zablotski, Eveline Lescrinier, Berthold Koletzko, Ulrich von Both, Katrin Hartmann

**Affiliations:** 1 Clinic of Small Animal Medicine, Centre for Clinical Veterinary Medicine, LMU Munich, 80539 Munich, Germany; k.zenger@medizinische-kleintierklinik.de (K.Z.); s.felten@medizinische-kleintierklinik.de (S.F.); n.bergmann@medizinische-kleintierklinik.de (M.B.); r.dorsch@medizinische-kleintierklinik.de (R.D.); Y.Zablotski@med.vetmed.uni-muenchen.de (Y.Z.); hartmann@lmu.de (K.H.); 2 Division of Paediatric Infectious Diseases, Dr. von Hauner Children’s Hospital, University Hospital, LMU Munich, 80337 Munich, Germany; Martin.Alberer@lrz.uni-muenchen.de (M.A.); Laura.Kolberg@med.uni-muenchen.de (L.K.); Ulrich.von.Both@med.uni-muenchen.de (U.v.B.); 3 Section of Clinical and Comparative Neuropathology, Institute of Veterinary Pathology, Centre for Clinical Veterinary Medicine, LMU Munich, 80539 Munich, Germany; kaspar.matiasek@neuropathologie.de; 4 Clinical Laboratory, Department of Clinical Diagnostics and Services, and Center for Clinical Studies, Vetsuisse Faculty, University of Zurich, CH-8057 Zurich, Switzerland; rhofmann@vetclinics.uzh.ch (R.H.-L.); mmeli@vetclinics.uzh.ch (M.L.M.); aspiri@vetclinics.uzh.ch (A.M.S.); 5 Department Paediatrics, Division Metabolic and Nutritional Medicine, Dr. von Hauner Children’s Hospital, University Hospital, LMU Munich, 80337 Munich, Germany; Jeannie.Horak@med.uni-muenchen.de (J.H.); Berthold.Koletzko@med.uni-muenchen.de (B.K.); 6 Institute of Novel and Emerging Infectious Diseases, Friedrich-Loeffler-Institut, Greifswald-Insel Riems, 17493 Greifswald, Germany; Saskia.Weber@fli.de (S.W.); Cora.Holicki@fli.de (C.M.H.); Martin.Groschup@fli.de (M.H.G.); 7 German Center for Infection Research (DZIF), Partner Site Hamburg-Luebeck-Borstel-Riems, Greifswald-Insel Riems, 17493 Greifswald, Germany; 8 Medicinal Chemistry, KU Leuven, Rega Institute for Medical Research, 3000 Leuven, Belgium; eveline.lescrinier@kuleuven.be; 9 German Center for Infection Research (DZIF), Partner Site Munich, 80337 Munich, Germany

**Keywords:** FIP, feline coronavirus, FCoV, treatment, therapy, antiviral chemotherapy, Xraphconn^®^, GS-441524, Mutian

## Abstract

Feline infectious peritonitis (FIP) caused by feline coronavirus (FCoV) is a common dis-ease in cats, fatal if untreated, and no effective treatment is currently legally available. The aim of this study was to evaluate efficacy and toxicity of the multi-component drug Xraphconn^®^ in vitro and as oral treatment in cats with spontaneous FIP by examining survival rate, development of clinical and laboratory parameters, viral loads, anti-FCoV antibodies, and adverse effects. Mass spectrometry and nuclear magnetic resonance identified GS-441524 as an active component of Xraphconn^®^. Eighteen cats with FIP were prospectively followed up while being treated orally for 84 days. Values of key parameters on each examination day were compared to values before treatment initiation using linear mixed-effect models. Xraphconn^®^ displayed high virucidal activity in cell culture. All cats recovered with dramatic improvement of clinical and laboratory parameters and massive reduction in viral loads within the first few days of treatment without serious adverse effects. Oral treatment with Xraphconn^®^ containing GS-441524 was highly effective for FIP without causing serious adverse effects. This drug is an excellent option for the oral treatment of FIP and should be trialed as potential effective treatment option for other severe coronavirus-associated diseases across species.

## 1. Introduction

Coronaviruses comprise a large family of RNA viruses that infect a wide variety of mammalian and avian hosts, causing severe disease in some of them [1,2,3]. Their high diversity causes the continuous emergence of new coronavirus variants that can have changed target cell tropism and/or host spectrum and lead to interspecies or zoonotic transmission; recent examples of the latter are Severe Acute Respiratory Syndrome Coronavirus (SARS-CoV), Middle East Respiratory Syndrome Coronavirus (MERS-CoV), and the most recent Severe Acute Respiratory Syndrome Coronavirus 2 (SARS-CoV-2) causing Coronavirus Infectious Disease 2019 (COVID-19) [4,5,6,7]. The newly emerging coronavirus-associated diseases raise awareness of the potential risks of highly virulent coronavirus infections in humans with close contact to animals harboring coronaviruses [8]. Shifts in tissue or cell tropism resulting in changes in virulence are distinctive features of coronaviruses. The best prototypic example for such a virulence change is the feline coronavirus (FCoV), which occurs as two different biotypes [9,10,11,12]. Only a small proportion (7–14% in multi-cat environments) of cats infected with FCoV, which is very common among multi-cat populations, develops the fatal disease feline infectious peritonitis (FIP) [13], triggered by spontaneous mutation of FCoV, thus gaining tropism for monocytes/macrophages in individual cats [14,15,16,17,18,19]. All cats with FIP either die or have to be euthanized without availability of effective treatment. The median survival time of untreated cats is only eight to nine days [9,20]. Previous controlled treatment trials with antiviral compounds either showed limited efficacy, such as the use of feline interferon-omega [9], or were too toxic for cats, such as ribavirin [21,22,23]. In most countries, such as in Europe and the United States, there is currently no effective licensed treatment option for cats with FIP, although unlicensed compounds have been imported and used by cat owners. It is important that veterinarians consult their local regulations and laws before embarking on the use of any unlicensed antiviral agents for the treatment of FIP due to variation in laws governing the veterinary profession and veterinary medicines worldwide.

However, a few studies have been published recently in which compounds not yet licensed showed extraordinarily promising results [24,25,26,27]. The most recent promising treatment for cats with FIP is the use of the nucleoside analogue GS-441524 [24,25,28], which is the active form of the prodrug remdesivir [29]. Currently, remdesivir is only conditionally licensed to treat human patients with severe COVID-19 symptoms [30,31,32]. Cats in these prospective studies received GS-441524 subcutaneously for various time periods and owners were instructed by social media groups to inject the compounds likewise for a period of 12 weeks or longer. Subcutaneous injections over such a long time period are difficult to perform by owners, can be very painful for cats due to the low pH of these unlicensed and uncontrolled preparations, and moreover could be associated with feline injection site sarcomas (FISS) [33]. Thus, oral compounds would be advantageous. Indeed, one case of successful treatment of a single cat with FIP (showing ocular signs but no effusion) with an oral preparation called Mutian (Mutian 200, Nantong Biotechnology, Nantong, China) has been reported [26]. Mutian was administered orally every 24 h (q24h) for 50 days and the cat received additional symptomatic treatment as well as feline interferon-omega. Within a few weeks of treatment, the cat showed marked gain in body weight and improvement of ocular signs as well as hematological and biochemical parameters. In two recent retrospective studies, cats were also treated with oral antivirals. In one, a large-scale online survey was addressed to cat owners who treated their cats with suspected FIP with an antiviral drug to collect their experiences, e.g., improvement of symptoms, potential relapses, and supportive therapy during the treatment [34]. In the other retrospective study from China, the disease course of 127 cats with suspected FIP was followed up; of these, 24 were treated with GS-441524 (although the route of administration was not mentioned) and only one cat relapsed [35]. However, to the authors’ knowledge, there are no published prospective studies on the oral treatment of cats with highly suspected or confirmed FIP with antiviral compounds. If the efficacy of an oral treatment in cats with a deadly disease like FIP could be consistently proven, this could also open up new avenues for future research investigating its effect in other severe corona-virus-associated diseases, such as COVID-19. Furthermore, due to its similarities of clinical features, the FIP-cat model could serve as natural model for elucidating pathophysiological features and potential treatment approaches in severe systemic inflammatory coronavirus-associated syndromes [6].

Therefore, the aim of this study was to prospectively evaluate the efficacy of an oral multi-component drug, first in vitro and then as oral treatment, in cats with naturally occurring FIP in a clinical trial, by determining survival rate, improvement of clinical and laboratory parameters, reduction of viral loads, and measurement of anti-FCoV antibodies. Additionally, potential adverse effects were recorded and the chemical structure of the active ingredient within the multi-component drug was identified by means of structural chemistry analysis.

## 2. Materials and Methods

### 2.1. Compound

The compound Xraphconn^®^ was used for the in vitro study as well as in the cats. It was provided by Mutian Life Sciences Limited in 2 batches (Supplementary File S1). According to the tablet insert, the active ingredient (referred to as MT-0901 in the package insert) was available at a concentration of 2.5 mg or 10 mg tablet, respectively. Other ingredients according to the tablet inserts were, among other substances, Radix scrophulariae, Platycodon grandiflorum, Phyllostachys pubescens, Forsythia suspensa, and Anemarrhena asphodeloides.

### 2.2. In Vitro Efficacy of the Multi-Component Drug

Efficacy of Xraphconn^®^ was first evaluated in an established cell culture model. Crandell-Rees Feline Kidney (CRFK) cells (Collection of Cell Lines in Veterinary Medicine CCLV, Friedrich-Loeffler-Institut, Greifswald-Insel Riems, Germany) were grown and maintained in Eagle’s minimal essential medium (MEM; Biochrom, Berlin, Germany) supplemented with 10% fetal calf serum (FCS; Biochrom) and kept at 37 ± 1 °C under a 5% CO_2_ atmosphere. Feline coronavirus RVB-1259 (a serotype I FCoV strain) was propagated once in CRFK cells in MEM as described above.

For the application on cells, a tablet (50 mg) said to contian 2.5 mg of the active ingredient was crushed using a mortar and 5 mL MEM or dimethyl sulfoxide (DMSO) was added. Suspensions were cleared by syringe filtration (Millex-GP syringe filter, pore size 0.22 µm). Subsequently, a logarithmic dilution was performed with both suspensions (1:10 up to 1:100,000), mathematically corresponding to an active ingredient concentration ranging from 50 µg/mL to 5 ng/mL at the assumption that the yield of extraction was 100%.

After exposure of the cells to the different dilutions of the filtrated tablet suspension, cell viability measurement using an MTT (3-(4,5-dimethylthiazol-2-yl)-2,5-diphenyltetrazolium bromide, a tetrazole) assay (Cell Proliferation Kit; Roche, Basel, Switzerland) was performed. Briefly, CRFK cells were seeded on a 96-well plate and different compound dilutions were added after 24 h. Cells were incubated for another 24 or 48 h, respectively. Subsequently, 10 µL MTT was added and cells were incubated for another 4 h. After incubation, solubilization solution was added and the enzymatic reaction was measured spectrophotometrically on the next day.

To evaluate the efficacy of Xraphconn^®^ in vitro, CRFK overnight cell cultures were infected with FCoV at a multiplicity of infection (MOI) of 0.01. After infection, wells were incubated at 37 ± 1 °C under a 5% CO_2_ atmosphere for 60 min and then washed with phosphate-buffered saline (PBS). Fresh culture medium (MEM supplemented with 5% FCS) containing different Xraphconn^®^ dilution levels was added. Finally, supernatants were collected at 24 or 48 h post infection. All tests were carried out as quadruplicate measurements.

Total RNA was extracted from all supernatants using the Nucleo-MagVet kit (MachereyNagel, Düren, Germany) according to the manufacturer’s instructions in an elution volume of 100 µL. Feline coronavirus RNA was detected by reverse transcriptase polymerase chain reaction (RT-PCR) as described below. To calculate the half maximal effective concentration (EC_50_), the viral RNA load for virus-infected non-treated cells was set to 100% and RNA values obtained for treated cells were normalized to this value; EC_50_-values were calculated by a non-linear regression analysis using GraphPad Prism 9.0 (GraphPad Software, San Diego, CA, USA).

### 2.3. Patients of the Prospective In Vivo Study

**Figure 1:**
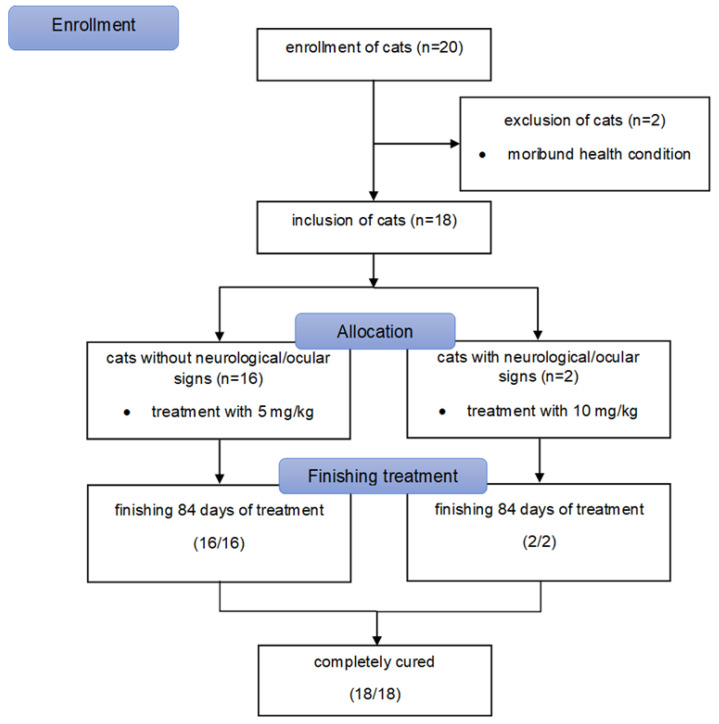
Flow diagram illustrating enrollment, inclusion, allocation process to high-dose treatment (10 mg/kg) for cats with neurological/ocular signs or low-dose treatment (5 mg/kg) for cats without neurological/ocular signs (based on the package inserts, Supplementary File S1), and outcome of cats in the study.

This study complied with the German guidelines for prospective studies and was approved by the Government of Upper Bavaria (reference number 55.2-2532.Vet_02-20-52) and by the ethical committee (reference number 261-19-03-2021) of the Centre for Clinical Veterinary Medicine of the LMU Munich. In addition, owners of the cats also gave their written consent to participate. Twenty cats were originally enrolled in the study (Figure 1). Inclusion criteria were (1) diagnosis of FIP, (2) body weight of at least 2 kg, (3) negative test results for feline immunodeficiency virus (FIV) and feline leukemia virus (FeLV) infection, and (4) absence of other severe diseases. Cats were excluded if (1) they were in a severe moribund or comatose condition at the time of presentation (as in such condition oral medications cannot be expected to be reliably absorbed), (2) it was not possible to administer tablets to the cats, or (3) owners were not compliant to either medicate their cats properly or to attend appointments.

A diagnosis of FIP was made (Table 1) if (1) virus was detected directly, either by immunohistochemistry detecting FCoV antigen within macrophages of altered organs [36,37] and/or by detection of a mutated strain of FCoV in effusion, blood, or an organ fine needle aspirate by a commercial RT-PCR and analysis targeting FCoV spike gene mutations leading to spike protein substitutions M1058L and S1060A (IDEXX laboratories, Ludwigsburg, Germany) in combination with (2) clinical and clinicopathological abnormalities considered typical for FIP [38] being present in the cats. Body weight was measured using a baby scale (AE Adam MTB 20 baby scale, Felde, Germany). The weight limit was set at 2 kg to comply with guidelines for blood volume sampling in cats according to the Gesellschaft für Versuchstierkunde—Society of Laboratory Animal Science [39]. Presence of anti-FIV antibodies and FeLV antigen was tested by the referring veterinarian or at presentation with either a point-of-care test or at a commercial laboratory. Absence of other severe diseases was determined by obtaining a complete history and physical examination as well as by performing an abdominal ultrasound in all cats on the day of entering the study; in addition, echocardiography was performed in cats with a thoracic effusion and a full neurological examination was performed in cats with neurological signs. Presence of mild disease (such as intestinal parasite infestation, otitis externa, rhinitis, chronic gingivostomatitis) did not lead to exclusion (Table 1). One cat had mild renal azotemia at the time of inclusion (Table 1). It was reasonable to suspect that these renal changes were caused by FIP and therefore the cat was included in the study. Of the 20 cats that were originally enrolled into the study, two cats were subsequently excluded due to their severe moribund health condition (Figure 1). Thus, 18 cats finally entered the study. Cats were then allocated to either high-dose treatment (10 mg/kg) if they had neurological/ocular (*n* = 2) or low-dose treatment (5 mg/kg) if they had no neurological/ocular signs (*n* = 16).

The age of the 18 cats (Table 1) ranged between 4.7 and 56.5 months (median 7.7 months) and 15 cats (83.3%) were younger than one year. Most cats (11/18; 61.1%) were European Shorthair (ESH); the others belonged to different breeds. The majority of cats was male (12/18; 66.6%) with 5 of them neutered. Of the female cats (6/18; 33.3%), 3 were neutered.

### 2.4. In Vivo Study Design

Cats were treated orally with Xraphconn^®^ for 84 days (day 0 to day 83). The dosage of the compound was chosen based on described studies with GS-441524 [24,28] and was based on the information of the tablet package insert on the concentration of the active component in the tablet. Thus, cats were treated with 5 mg/kg of the active component per os (PO) q24h if they had no neurological and/or ocular signs and at 10 mg/kg PO q24h if they had neurological and/or ocular signs. Dosage of Xraphconn^®^ tablets was adjusted daily to the determined weight and tablets were always administered at the same time on an empty stomach. Half an hour after tablet administration, food was provided to the cats.

All cats were hospitalized for the first 8 days (day 0 to day 7). During this time, cats were under intensive surveillance and medical care by board-certified specialists in internal medicine and intensive care for 24 h per day, and all diagnostic procedures, supportive measures, and symptomatic treatments were applied as necessary (Table 1). On day 0, before treatment with Xraphconn^®^, a history and complete physical examination as well as hematological and clinical chemistry parameters were obtained in each cat. Several parameters were evaluated during the first 8 days and at several time points during the rest of the treatment period (Table S1). This included physical examination (including measurement of body weight and determination of the modified Karnofsky’s score (Table S2) (day 0, 1, 2, 3, 4, 5, 6, 7, 14, 28, 56, 83), abdominal ultrasound (day 0, 4, 7, 14, 28, 56, 83), hematology and clinical chemistry (including symmetric dimethylarginine (SDMA) and serum amyloid A (SAA)) (day 0, 2, 4, 7, 14, 28, 56, 83), and measurement of blood viral load (day 0, 2, 4, 7, 14, 28, 56, 83), effusion viral load (in case of effusion accessible for drainage), and serum anti-FCoV antibodies (day 0, 7, 14, 28, 56, 83). As long as the cats had abdominal or thoracic effusions, abdominal or thoracic ultrasound and if possible abdominocentesis or thoracocentesis, respectively, were performed daily, and fluid was stored for determination of viral load.

The Karnofsky’s score modified for cats by Hartmann and Kuffer (1998) was used to evaluate the general condition and well-being of the cats (Table S2) using a classification from 0% (dead) to 50% (normal health condition) [40,41]. Hematology was performed using an automatic analyzer (Cell-Dyn 3500, Abott Laboratories, IL, USA). Differential blood count was additionally performed manually on blood smears exposed to Haema Quick Staining/Diff-Quick staining if hematology parameters were abnormal. Serum biochemistry parameters were measured by an automatic analyzer (Hitachi 911, Roche, Grenzach-Wyhlen, Germany), SDMA was analyzed at IDEXX Diavet AG (Bäch, Switzerland) using a high-throughput immunoassay, and SAA was determined using a latex agglutination turbidimetric immunoassay reaction (LZ Test SAA, Eiken Chemical Co., Ltd., Tokyo, Japan) on a cobas^®^ c 501 clinical chemistry analyzer (Roche Diagnostics AG, Rotkreuz, Switzerland).

Cats were discharged on day 7 (unless clinical condition required prolonged inpatient care), and owners continued daily administration of the study drug. Owners received personalized instructions, including videos, on how to administer the tablets. They were instructed to call in at any time in case of problems. For the remaining period, owners had to closely monitor their cats at home and remain in close contact with the study team. Cats were not allowed outdoors during the treatment period. However, 17/18 cats had between 1 and 9 partner cats (most of which were allowed to go outside) in the same household with close contact (Table 1). Owners had to keep a daily diary for recording their cats’ clinical parameters and body weight to guarantee optimal drug dosing at all times.

### 2.5. FCoV Viral Load in Blood and Effusion

Feline coronavirus RNA load was determined on several days in blood and effusion samples (as long as present). Samples were stored at −80 °C and analyzed collectively by RT-quantitative PCR (RT-qPCR). Viral total nucleic acids (TNA) were extracted from 200 μL of effusion or 100 μL ethylenediaminetetraacetic acid (EDTA) anticoagulated whole blood using the MagNa Pure 96 (Roche Diagnostics AG, Rotkreuz, Switzerland) and the MagNA Pure 96 DNA and Viral NA SV Kit (Roche Diagnostics AG, Risch-Rotkreuz, Switzerland) according to the manufacturers’ instructions with an elution volume of 100 µL. For all samples, the viral nucleic acid (NA) plasma external lysis SV 4.0 protocol was applied. For each batch of extractions, negative controls were run in parallel to check for cross-contamination.

A previously published RT-qPCR assay was used to detect the FCoV 7b gene [42]. The methods were adapted as follows: primer and probes [42] were used with the AgPath-ID^TM^ One-step RT-PCR kit (Applied Biosystems, Rotkreuz, Switzerland). The mastermix consisted of 1X RT-PCR buffer, 1.0 μL Array Script reverse transcriptase and AmpliTaq Gold DNA polymerase, 300 nM forward primer (FCoV2), 900 nM reverse primer (FCoV1), 300 nM probe (FCoVp), and nuclease-free H_2_O was added to a final volume of 20 μL. All RT-qPCR assays were run with 5 μL of TNA in a final volume of 25 μL. Positive and negative controls were run in parallel using a ABI 7500 Fast instrument (Applied Biosystems). All samples were tested for absence of inhibition. A FCoV RNA standard curve was run in parallel to determine the viral RNA copy number.

### 2.6. Anti-FCoV Antibodies

Antibodies against FCoV were measured on days 0, 7, 14, 28, 56, 83. Serum samples were stored at −80 °C and analyzed by an indirect immunofluorescence assay (IFA) as previously described [43,44,45,46]. Briefly, cat samples were tested at dilutions of 1:25, 1:100, 1:400, 1:1600, and 1:6400. The fluorescein isothiocyanate (FITC) conjugated secondary antibody (rabbit anti cat IgG (H + L) (Nordic-MUbio, Sustern, Netherlands; LuBio Sience GmBH, Luzern, Switzerland) was diluted at 1:40. Slides were prepared using porcine kidney cells (PD-5 cells) infected with transmissible gastroenteritis virus (TGEV, Purdue strain). The antigen preparations were tested for the absence of contaminating viruses by RT-qPCR and qPCR as previously described [45]. A positive control (aliquoted serum sample of a FCoV-antibody-positive field cat) and a negative control (aliquoted serum from a specific pathogen free FCoV-antibody-negative cat) were run with each slide.

### 2.7. Characterization of the Active Ingredient in the Multi-Component Drug

Characterization of the active ingredient was performed by mass spectrometry (MS) and nuclear magnetic resonance (NMR). For details regarding these analyses, refer to the supporting information (Supplementary Text S1).

### 2.8. Data Analysis

Data were analyzed using R statistical language (version 4.0.3; R Core Team, 2020). Due to the repeated measures for an individual animal on multiple days, all variables were evaluated using linear mixed-effects models, with an individual animal as a random effect. The following model assumptions were always checked: (1) the normality of residuals was evaluated by the Shapiro–Wilk normality test, (2) the homogeneity of variances was evaluated with Levene’s test, (3) the heteroscedasticity (constancy of error variance) was evaluated with Breusch-Pagan test. In case assumptions were violated, a robust linear mixed-effects regression (RLMER) was applied; RLMER computes weighted estimates via Design Adaptive Scale approach and thus, solves heteroskedastic and non-normally distributed residuals by assigning lower weights to outliers and other contaminations. All contrasts (differences) between particular days were assessed after model-fitting by the estimated least-squares marginal means with the Bonferroni *p*-value correction for multiple comparisons. For comparison of the number of cats with effusion per day, pairwise Fisher’s tests were performed also with the Bonferroni *p*-value adjustment for multiple testing. Results with a *p*-value ≤ 0.05 were considered statistically significant. A nonparametric bootstrap with replacements that does not assume normality was used for obtaining and visualizing means and 95% confidence limits of blood and effusion viral loads.

## 3. Results

### 3.1. Efficacy of the Multi-Component Drug In Vitro

**Figure 2:**
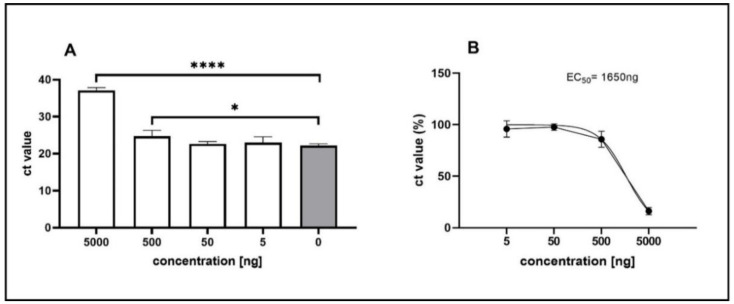
Feline coronavirus (FCoV) replication inhibition by Xraphconn^®^. (**A**) FCoV cycle threshold (ct) values in supernatants collected from infected Crandell-Rees Feline Kidney (CRFK) cells (multiplicity of infection (MOI = 0.01) treated with the indicated active compound concentrations were collected at 24 h post infection (*n* = 4). Significance levels compared to the results for untreated cells were determined by the Bonferroni’s multiple comparisons test and are indicated as follows: *, *p* ≤ 0.05; ****, *p* < 0.0001. (**B**) Data from four biological replicates were used to calculate the half maximal effective concentration (EC_50_) value by non-linear regression analysis.

The cytotoxic effect of the multi-component drug Xraphconn^®^ solubilized in MEM or DMSO was examined on CRFK cells using an MTT assay. After 24 and 48 h of treatment, the Xraphconn^®^ solution exhibited no major cytotoxicity at a dilution of 1:100, independent of the applied solvent. Therefore, Xraphconn^®^ solubilized in MEM at 1:100 and successive log dilutions were used for the antiviral efficacy testing. For this purpose, FCoV (MOI of 0.01) replication in Xraphconn^®^-treated and non-treated CRFK cells was measured by RT-qPCR, and FCoV replication inhibition by Xraphconn^®^ (concentration 1:100) was significant, as viral RNA levels in treated cells were drastically reduced compared to the non-treated controls. This dilution corresponds to a concentration of 5 µg/mL of the active compound in the solution. For FCoV-infected CRFK cells, the calculated EC_50_-value of the Xraphconn^®^ solution was 1.65 µg/mL (Figure 2).

### 3.2. Efficacy of the Multi-Component Drug in Cats With FIP

All 18 cats completely recovered clinically within the 84 days of treatment. No relapse occurred, and at the time of publication (99 days after the last cat had finished its treatment course), all cats were still alive. With one exception, all cats were healthy and asymptomatic at the end of the treatment period. The only cat that showed any clinical signs at the end of treatment, as well as at the time of publication, was the cat presenting with renal azotemia at time of inclusion in the study (cat 3). This cat recovered clinically from all other signs related to FIP, but developed unilateral renal mineralization, which was first observed on day 21 of treatment. Renal azotemia improved with fluid therapy and overall remained stable during the treatment period. On day 168, three months after the end of treatment, the cat had developed unilateral ureteral obstruction with hydronephrosis and mild ascites, which most likely occurred secondary to the obstruction and was unrelated to FIP.

**Figure 3:**
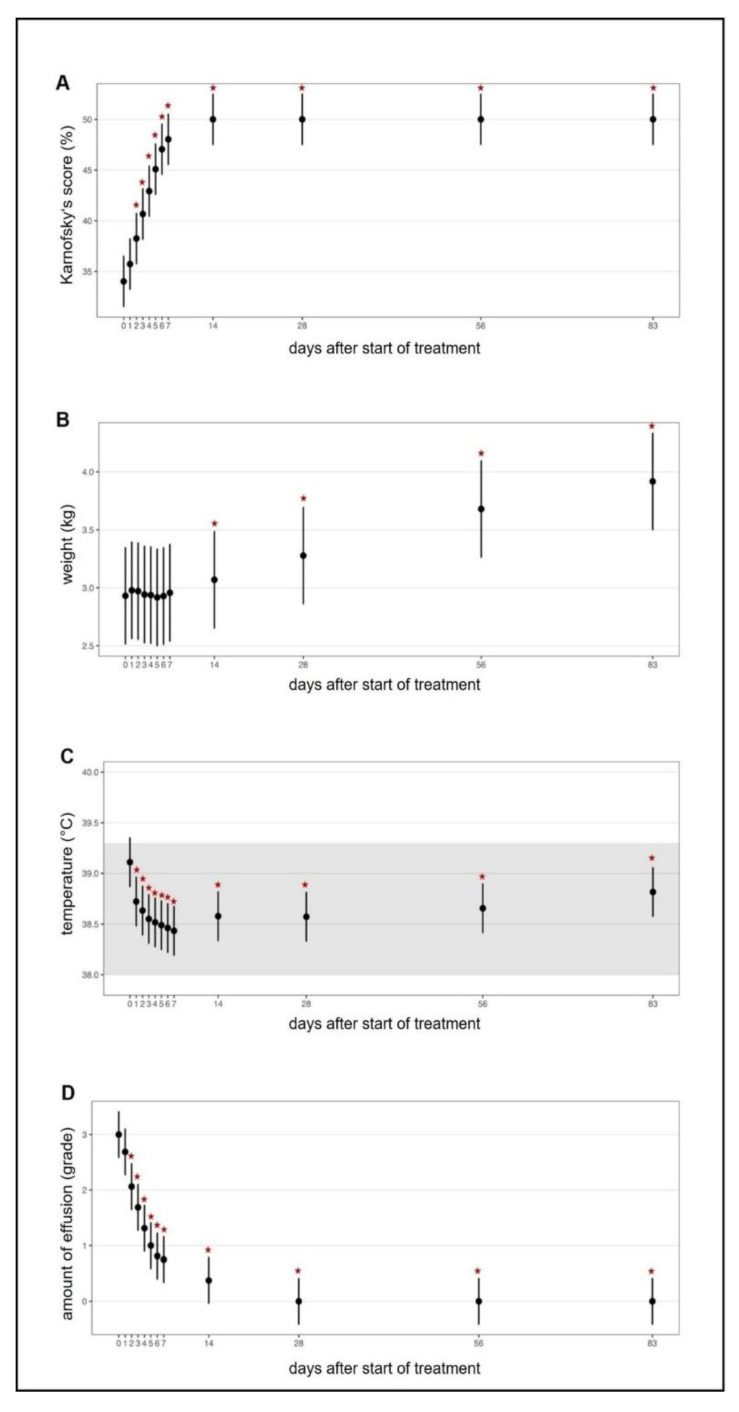
Timeline visualizing improvement of clinical parameters throughout the study course. Figures show average predictive values and 95% confidence intervals of each parameter. Grey shading marks the reference ranges of the parameters. Red asterisks mark significant difference (*p* ≤ 0.05) of the parameters on different days of treatment when compared to day 0 (before treatment) measured by a linear mixed-effects model (for temperature) and by robust linear mixed-effects models. (**A**) Karnofsky’s score modified for cats. (**B**) Body weight. (**C**) Body temperature. (**D**) Amount of effusion subjectively evaluated during abdominal/thoracic ultrasound and paracentesis (grades 0 (no fluid) to 4 (massive effusion)).

**Figure 4:**
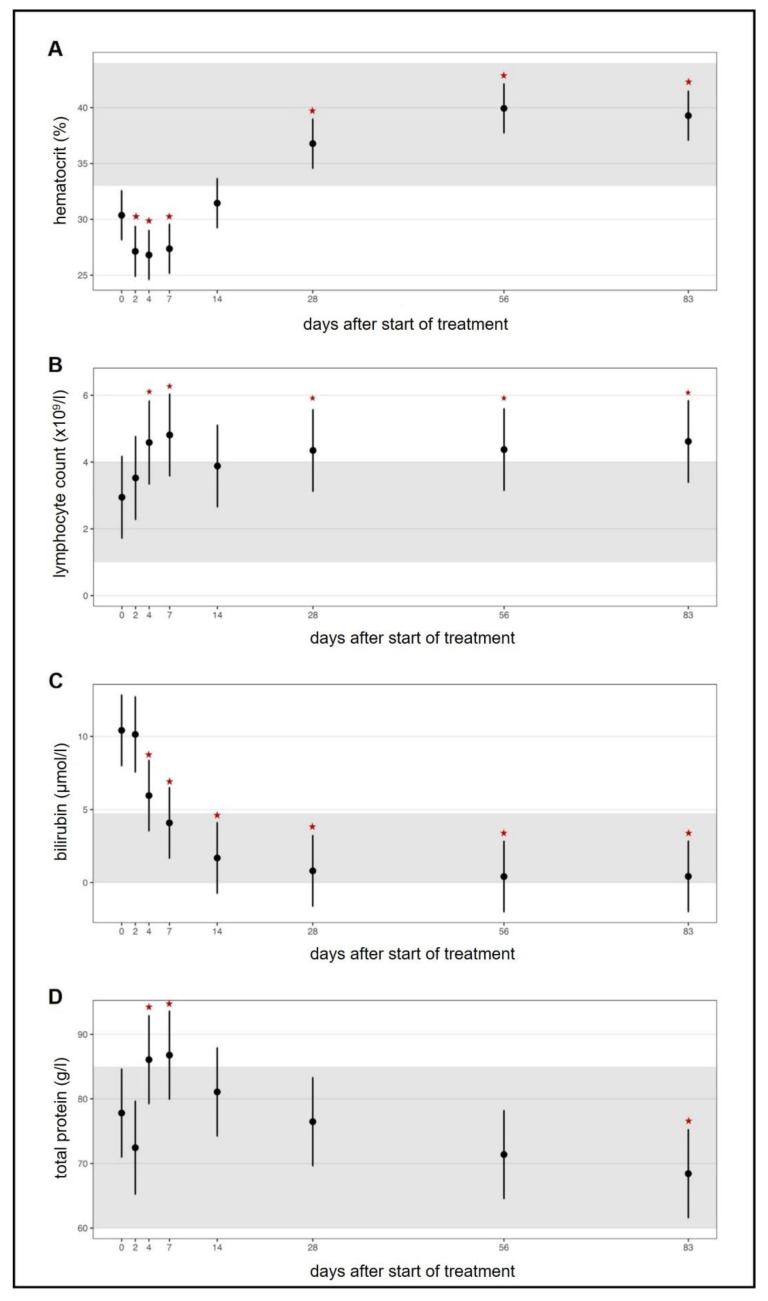

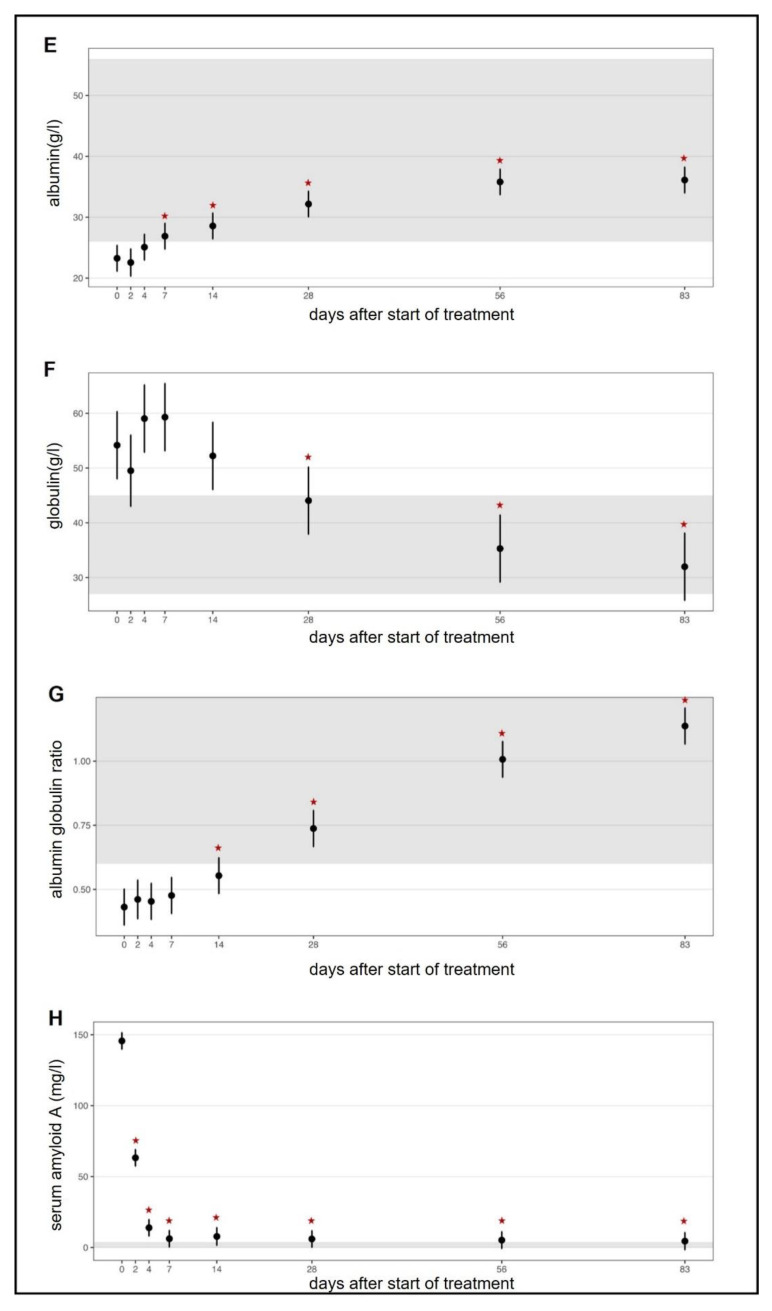
Timeline visualizing improvement of clinicopathological parameters throughout the study course. Figures show average predictive values and 95% confidence intervals of each parameter. Grey shading marks the reference ranges of the parameters. Red asterisks mark significant difference (*p* ≤ 0.05) of the parameters on different days of treatment when compared to day 0 (before treatment) measured by a linear mixed-effects model (for albumin) and by robust linear mixed-effects models. (**A**) Hematocrit. (**B**) Lymphocyte count. (**C**) Bilirubin concentration. (**D**) Total protein concentration. (**E**) Albumin concentration. (**F**) Globulin concentration. (**G**) Albumin/globulin ratio. (**H**) Serum amyloid A (SAA) concentration.

**Figure 5:**
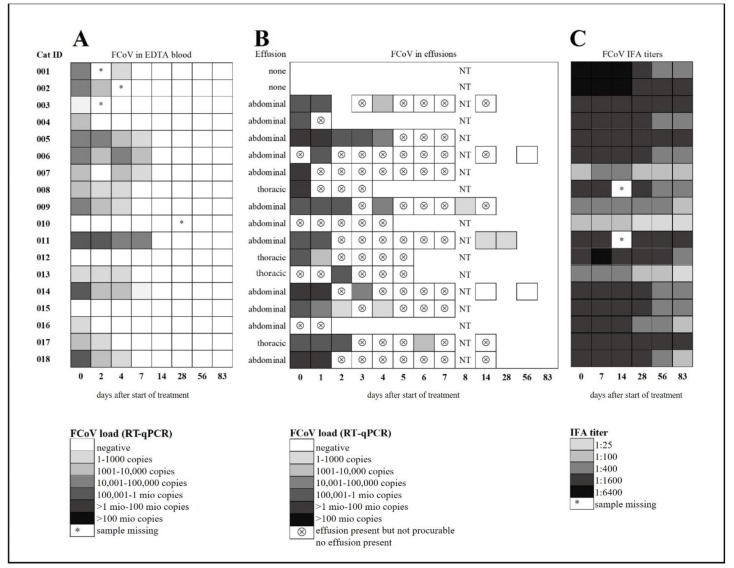
Feline coronavirus (FCoV) viral RNA loads in blood and effusion samples and serum anti-FCoV antibody titre measurements. (**A**) FCoV RNA loads in EDTA anticoagulated blood. (**B**) FCoV RNA loads in effusions. (**C**) Serum anti-FCoV antibody titres. FCoV RNA loads were determined by quantitative reverse transcriptase polymerase chain reaction (RT-qPCR) (**A**,**B**). Antibody titers were determined by indirect immunofluorescence assay (IFA). NT, not tested.

**Figure 6:**
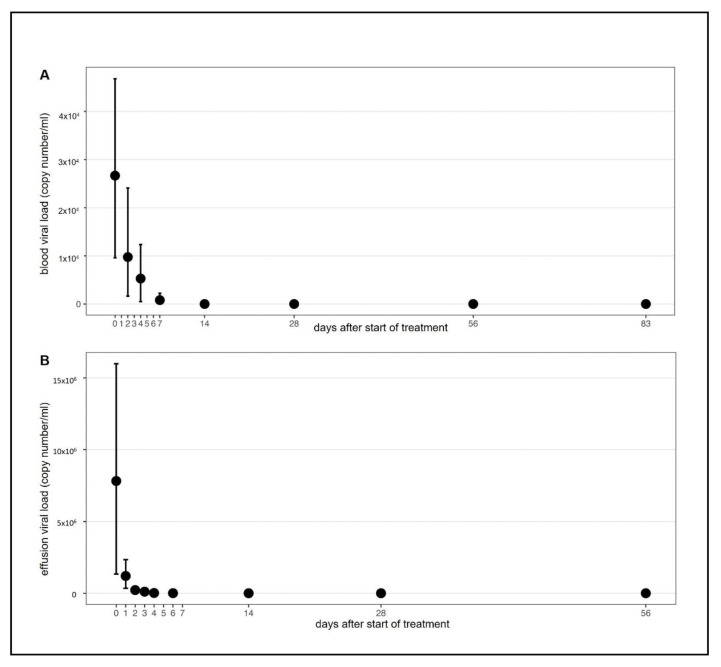
Viral load in blood and effusion throughout the study course. Figures show visualization of data using nonparametric bootstraps. (**A**) FCoV RNA loads in EDTA anticoagulated blood. (**B**) FCoV RNA loads in effusions.

In all treated cats, clinical and laboratory parameters improved constantly and significantly during the treatment (Figure 3, Figure 4, Figure 5 and Figure S1). Of the 18 cats, 17 were discharged from the hospital after the first 7 seven days with a modified Karnofsky’s score of at least 45% (with 50% being equivalent to completely healthy) (Figure 3A); one cat had to stay hospitalized for 11 days due to the development of pyothorax. All cats gained body weight rapidly (Figure 3B) and had normal body temperatures soon after treatment initiation (Figure 3C) and the amount of effusion decreased markedly (Figure 3D), as did the number of cats that still had effusion (Figure S1). In addition, all clinicopathological parameters (hematological and clinical chemistry parameters including SAA) improved steadily (Figure 4A–H) with significant differences in the parameters by day 1 to day 28 when compared to day 0 (before treatment). Remarkably, in most of the cats (15/18), viral RNA could be detected in blood before treatment, but blood viral loads decreased massively in all cats by day two to four after treatment initiation (Figure 5A and Figure 6A). By day 14, viral RNA was no longer detected in the blood of the cats (Figure 5A and Figure 6A), indicating that all had cleared detectable viremia. All accessible effusions tested RT-qPCR-positive at the start of the study (Figure 5B and Figure 6B). Later on, viral RNA was detectable in all but three effusion samples (Figure 5B). However, these three cats had smaller volumes of effusion collected and evaluated by RT-qPCR. All cats had serum anti-FCoV antibodies at the initiation of the study, most of them with high antibody titers (14/18 with titers ≥1:1600). Anti-FCoV antibody titers declined in 14/18 cats, in some cats starting as early as 28 days after the initiation of treatment (Figure 5C and Figure S2). In none of the cats was an antibody titer increase observed.

### 3.3. Adverse Effects of the Multi-Comoonent Drug in Cats

No serious adverse effects were observed during Xraphconn^®^ treatment (Table 2) and therefore in none of the cats did treatment have to be discontinued. Mild Heinz body anemia (with 19.8% of red blood cells containing Heinz bodies, hematocrit 32.5% (reference range: 33–44%)) was observed in one cat on the last day of the Xraphconn^®^ treatment (day 83). The cat was treated with S-adenosyl-methionine (Table 1) and the hematocrit normalized (to 34.7% on day 100) and Heinz bodies decreased (to 8.8% on day 126). Lymphocytosis was seen in 14/18 (77.7%) cats; three of those already had moderate, and two of those severe, lymphocytosis before treatment initiation (Table 2). In most of the cats, however, lymphocytosis was only mild to moderate. One cat (before treatment, the cat had a lymphocyte count at the lower end of the reference range (1.1 × 10^9^/L)), however, developed a massive lymphocytosis on day 83 (40.7 × 10^9^/L). Cytology of a blood smear revealed a high number of predominantly small lymphocytes, isolated large or reactive lymphocytes with dark blue cytoplasm. To rule out chronic lymphocytic leukemia, a PCR for Antigen Receptor Rearrangements (PARR) analysis was performed, which was consistent with a reactive lymphoid cell population. The majority of cats (11/18; 61.1%) developed eosinophilia during the treatment period, but in all cats this was only mild (<2.0 × 10^9^/L). Fecal examinations were performed in 10/11 cats with eosinophilia to exclude parasite infestation. In one cat, *Giardia* spp. infection was present before start of the Xraphconn^®^ treatment, and the cat was treated with fenbendazole; in another cat, *Giardia* spp. infection was detected by fecal examination on day 56 (this cat had no eosinophilia; fecal examination was performed because the partner cat suffered from *Giardia* spp. infection). An increase in liver enzyme activity was noted in 11/18 cats between day 2 and day 83, but was mostly only mild to moderate. An increase of alanine aminotransferase (ALT) activity was observed in 7/18 (38.9%) cats; of those, two cats had a severe ALT activity increase and were treated with silymarin. An increase in alkaline phosphatase was seen in 8/18 cats, but was only mild (seven cats) to moderate (one cat). No renal toxicity was detected. With one exception, SDMA remained within the reference range throughout the whole treatment period. One cat had renal azotemia, which was already present before treatment initiation with increased creatinine, urea, and SDMA. None of the cats developed azotemia or showed an increase in creatinine of more than 26.4 µmol/L in the non-azotemic range within 48 h and none of the cats developed oliguria, and thus, there was no evidence of acute renal toxicity [47]. Mild prerenal azotemia (with isolated urea concentration elevation between 11.6–12.7 mmol/L) was detected in four cats, but those cats had not been fasted before blood sampling.

### 3.4. Characterization of the Main Active Ingredient in the Multi-Component Drug 

**Figure 7:**
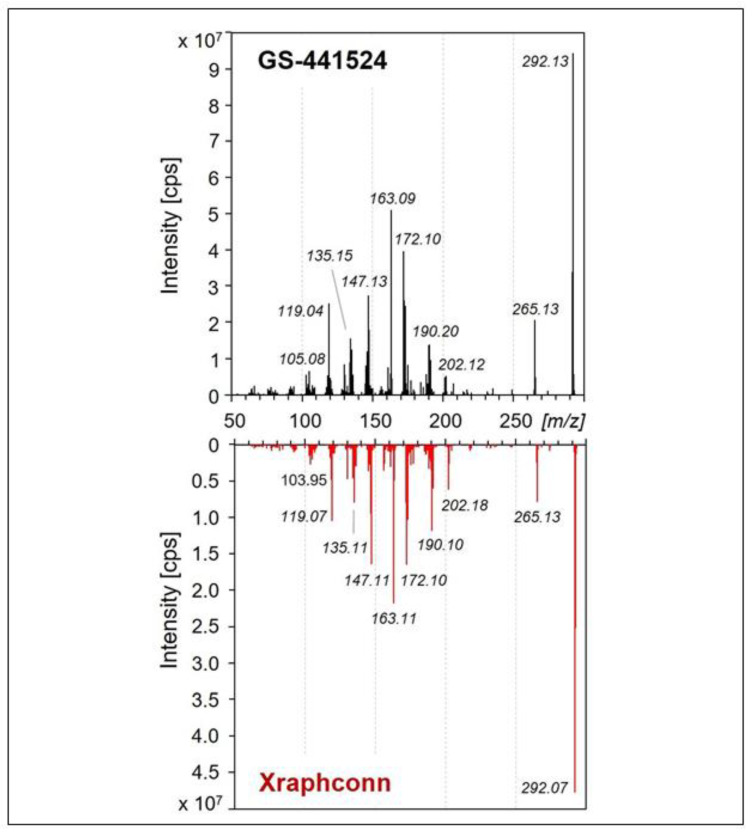
Comparison of the Ultra-High-Performance-Liquid Chromatography Electro-Spray QTRAP Mass Spectrometry (UHPLC-ESI-QTRAP-MS/MS) spectra of GS-441524 and the active component of Xraphconn^®^ extracted from the tablet. The compounds in both test solutions were not only isobaric with [M + H]+ m/z 292.1040, but exhibited identical fragmentation spectra. Mass spectra were generated at a collision energy of 50 eV with positive ionization using the Multiple Reaction Monitoring with Information Dependent Acquisition and Enhanced Product Ion (MRM-IDA-EPI) scan mode. Cps, counts per second.

Evaluation of the structure of the active component in Xraphconn^®^ by Ultra-High-Performance-Liquid Chromatography Electro-Spray QTRAP Mass Spectrometry (UHPLC-ESI-QTRAP-MS/MS) using Multiple Reaction Monitoring with Information Dependent Acquisition and Enhanced Product Ion Scan (MRM-IDA-EPI scan) provided a MS/MS fragment pattern that was practically identical to that of a control measurement performed with GS-441524 (Figure 7).

As LC-MS/MS could not distinguish between a furanose ring of GS-441524 and a pyranose ring and a ring opening during collision-induced decay (CID) would provide very similar MS-fragmentation patterns of the two compounds, further structure elucidation could only be performed via NMR spectroscopy.

**Figure 8:**
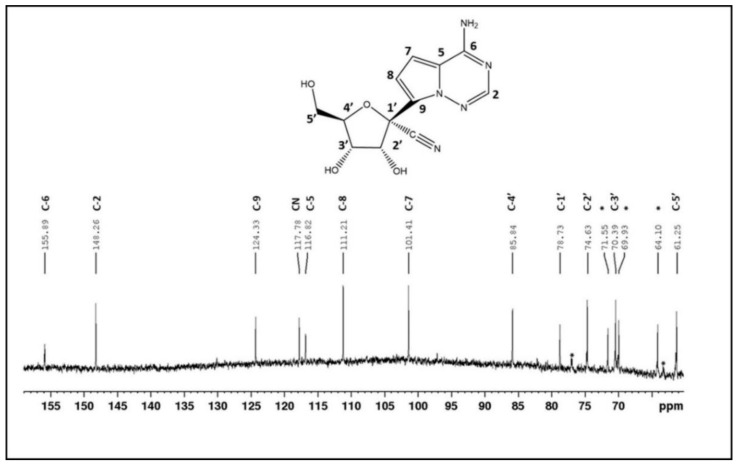
13C spectrum of the analyzed sample. Labels refer to the assignment of carbons in the active component of Xraphconn^®^ depicted above. All signals above 100 ppm belong to the cyano-group and nucleobase in the identified compound. Some additional signals of uncharacterized impurities were observed below 80 ppm (indicated with *). Ppm, parts per million.

In NMR, starting from a 2D correlated spectroscopy (2D-COSY) spectrum [48], all non-exchangeable protons and exchangeable hydroxyl protons of the major compound in the sample could be assigned. The observed HO-5′ (with COSY cross peaks to both H-5′ protons) and absence of HO-4′ are characteristic for a furanose ring, while in a pyranose ring, the opposite is expected. The Heteronuclear Single Quantum Coherence (HSQC) spectrum [49] provided assignment of carbon signals for CH2 and CH groups in the molecule, exploiting J-coupling interaction over the proton-carbon chemical bond. Quaternary carbons were assigned by 3-bond J-coupling interaction between protons and carbons in the Heteronuclear Multiple Bond Correlation (HMBC) spectrum [50]. The cross-peak of H-2′ to CN in this HMBC spectrum allowed to unambiguously determine the position of the cyano-group in the molecule. Obtained 13C chemical shifts (Figure 8) and patterns of 1H signals (Table S3) correspond to what is reported on GS-441524 [51]. Differences in 1H chemical shifts can be attributed to different sample conditions for pure GS-441524 in fully deuterated dimethyl sulfoxide (DMSO-d6) relative to the extract of Xraphconn^®^ in a DMSO-d6/D2O mixture including salts and other impurities.

## 4. Discussion

In this first prospective controlled treatment trial using an oral antiviral compound in field cats with FIP, it could be clearly demonstrated that oral treatment with GS-441524 -containing Xraphconn^®^ displays a striking efficacy. All 18 treated cats showed a very swift response to the treatment with rapid improvement of clinical and clinicopathological parameters leading to full and relapse-free recovery. Without treatment, virtually all cats suffering from FIP die, making FIP one of the most lethal diagnoses in the cat population, instead, the survival rate in the present study for the 18 cats was 100% at the time of publication. In addition, viral loads decreased in both blood and effusion (where present and available for RT-qPCR) within a short period of time after treatment initiation, and none of the cats was blood FCoV RNA-positive after day 14 demonstrating an enormous response to treatment. Also, antibody titers decreased significantly during therapy. The in vivo efficacy also mirrors the in vitro data, where GS-441524 containing Xraphconn^®^ also showed excellent efficacy and induced a rapid decrease of viral loads in FCoV-infected CRFK cells.

All clinicopathological parameters that are typically altered in cats with FIP improved significantly within a few days [37]. Typically, total protein, globulin, and bilirubin concentrations are elevated in cats with FIP, while albumin concentration, albumin/globulin ratio, hematocrit, and lymphocyte counts are decreased [52,53]. All these values normalized within a short period of time with Xraphconn^®^ treatment. Body temperature was elevated in some cats, but decreased to the reference range during the first few days in the clinic. However, temperature was generally higher during control visits when compared to the first days in the clinic, which might be explained by the stress during transportation. Also, SAA concentrations rapidly and significantly decreased upon treatment in all cats. SAA is a major acute phase protein in cats, and SAA expression has been shown to be increased in cats with FIP, indicating the presence of a severe inflammatory response [54,55,56,57]. The marked reduction in SAA concentrations in response to treatment reflects the clinical efficacy of the multi-component drug Xraphconn^®^ and suggests a remarkable attenuation of the hyperinflammatory response seen in cats with FIP.

Structural chemical analysis to identify the active components of the multi-compound drug by using mass spectrometry and NMR identified GS-441524 as the single adenine C-nucleoside ribose analogue extracted from Xraphconn^®^. No other similar nucleoside analogues were detected, although it cannot be excluded that there might be additional active ingredients present in Xraphconn^®^.

Although a very good efficacy of GS-441524 upon subcutaneous administration has been demonstrated in an experimental study and in cats in the field before [25,28], the present study demonstrated an even higher efficacy, as all of the 18 treated cats were apparently cured when the drug was administered orally. There are several potential reasons for these favorable results. First, additional substances to the GS-441524, such as herbal preparations, in the multi-component drug Xraphconn^®^ might increase efficacy with some of these substances exhibiting synergistic effects. Second, only cats with a confirmed diagnosis or at least very likely diagnosis of FIP were included in the present study and therefore, there was little chance that cats with other severe diseases were mistakenly treated with the drug. Third, all cats received intensive medical care 24/7 as well as symptomatic treatment, especially during the first critical seven days, including in-depth diagnostic procedures and individualized supportive treatment in a highly specialized university hospital.

This is the first time that oral (instead of subcutaneous) treatment was applied to cats with FIP in a controlled study. In the past, oral treatment was used successfully in a case report in one cat with neurological FIP [26]. The study of Jones and coworkers (2021) “unlicensed GS-441524-like antiviral therapy can be effective for at-home treatment of feline infectious peritonitis” [34] was an online survey study that drew its findings from testimonials from private owners who had provided independent and non-standardized as well as non-supervised therapy to their cats. Similarly, the study of Yin and coworkers (2021) “a retrospective study of clinical and laboratory features and treatment on cats highly suspected of feline infectious peritonitis in Wuhan, China” [35], was a retrospective study and did not have any prospective aspect. Thus, these studies were not prospective and therefore are not comparable to the current study.

Oral treatment has several advantages. In previous studies with subcutaneous injections of GS-441524, more than half of the cats developed injection site reactions [25]. In such inflammatory reactions could trigger development of FISS. In addition, handling of subcutaneous injections is difficult and challenging for owners and can be very painful for the cats causing injection reactions. In the present study, all 18 cats treated orally remained cooperative throughout the entire treatment period and the owners were able to successfully administer the tablets.

The study protocol set a fixed dose for each participating cat (10 mg/kg for cats with neurological/ocular signs and 5 mg/kg for cats without neurological/ocular signs). The package insert specified the concentration of the active component within the tablets and the dosages applied to cats were based on this information and on the previous published studies [24,28]. However, the exact amount of GS-441524 within the Xraphconn^®^ tablets was not verified in the present study, but all cats were apparently cured and no major adverse effects were reported, it has to be assumed that the dose given in the package insert was correct. Individualized dosing of cats, as sometimes recommended by social media groups, based on laboratory parameters, such as globulin concentrations, did not appear necessary or indicated in the current study. In addition, a standardized additional symptomatic therapy for each cat as advised by social media groups, e.g., tablets for liver protection, was not necessary. The present study clearly demonstrates the importance of adapting symptomatic treatment according to the problems and needs of the individual cats. Unrelated problems can occur, such as a renal mineralization in one of the cats in the current study, which would not have been detected if owners had treated their cats at home without veterinary supervision, which can lead to significant problems, independent of FIP, without adequate diagnosis and therapy. Thus, FIP treatment should not be left in the hands of owners alone, and legal approval of the drug in veterinary medicine is urgently needed. Whether a shorter treatment duration would be possible and successful, and to aid in the reduction of high costs, remains to be determined. In the present study, no cat was viremic by day 14 after starting treatment and viral levels in effusions were similarly reduced. It might therefore be interesting to investigate the efficacy of a shorter treatment period in a well-controlled study.

Two cats had to be excluded from the study due to their severe moribund condition. In the present study, cats were only included after extensive efforts were made to obtain a diagnosis of FIP. This was performed by applying an individualized multi-step diagnostic approach, including extensive physical examination, basic clinicopathological data, diagnostic imaging, and methods for direct virus detection (immunohistochemistry and/or spike gene mutation analysis with RT-PCR). A diagnosis of FIP cannot be obtained easily by one diagnostic test, since clinical and pathological abnormalities and imaging findings are not pathognomonic, and the detection of viral RNA by RT-PCR with or without mutation analysis, is not diagnostic for FIP [36]. Thus, a multimodal diagnostic approach is highly advisable; however, this is time-consuming, and disease progression in the meantime might preclude treatment success in some cats with severe disease. Nevertheless, in the face of cost and emergence of viral drug resistance, the aim should still be to be as confident as possible that a cat is truly suffering from FIP before starting antiviral treatment, and this can only be done by veterinarians, again highlighting the importance of having veterinary involvement in FIP diagnosis and treatment.

The viral RNA loads in accessible abdominal or thoracic effusions decreased in all cats over the study period, and looking at the blood viral RNA loads, it is assumed that viral RNA was also cleared from the thoracic and/or abdominal compartments. The Xraphconn^®^ treatment was therefore beneficial in reducing viral RNA loads in effusions and blood and cured abdominal and/or thoracic effusions in cats with FIP. A remarkably high percentage of cats with FIP in this study (83%) were viral RNA-positive in blood upon entering the study. In other studies in cats with confirmed FIP, detection of viral RNA in blood was rather a rare event [53,58,59,60,61]. Thus, the notion that viremia will likely usually be over by the time clinical signs of FIP appear [42], has to be reconsidered in view of the current data. Most cats in the present study with FIP had remarkably high antibody titers, which decreased in most but not all cats over time.

The adverse effects reported with the Xraphconn^®^ treatment in the present study were considered to be acceptable and not serious, and treatment did not need to be discontinued in any of the cats. No gastrointestinal signs were caused by oral administration. Previous studies using GS-441524 have mainly reported adverse effects related to subcutaneous injection (e.g., pain reactions, superficial skin lesions, skin ulceration). A decrease in hematocrit, total protein, and albumin during the first days of treatment in the clinic likely occurred due to blood sampling and might be a dilutional effect due to fluid therapy, but was fully reversible. The most pronounced adverse effect seen in the present study was a lymphocytosis that occurred in 14/18 cats. Significant abnormalities in hematological parameters have not been reported as adverse effects in association with GS-441524 treatment in field cats so far [24,25,26,28]; however, a marked increase in the lymphocyte count was also seen after treatment of cats with experimental FIP [28]. Lymphocytosis could have been caused by excitement to which cats are very sensitive, or by antigen stimulation. While younger cats generally have higher lymphocyte counts, the changes were too pronounced to be explained only by young age. The changes observed for lymphocyte counts in response to treatment appear to be of particular interest. Though this study did not aim to characterize the host immune response in detail, it is interesting to observe that mild to moderate lymphocytosis was seen in >75% of cats following treatment initiation, while SAA concentrations displayed a rapid and sustainable decline in all participating animals. One cat developed a massive lymphocytosis on day 83 (40.7 × 10^9^/L) and PARR-based exclusion of clonality and thus malignancy suggested a reactive lymphoid population. To some extent, these lymphocyte responses remind the clinician of features seen in Immune-Reconstitution Inflammatory Syndrome (IRIS) [62]. One could speculate that cytokines produced by monocytes upon FCoV infection could cause both a reduction of lymphocytes and an impaired immune response to FCoV. Lymphopenia is a common feature [53,63] and negative prognostic factor [53] in cats with FIP, caused by increased apoptosis of B and T cells induced by tumor necrosis factor alpha expression [64,65,66,67,68]. As a consequence, a rebound in the immune response could occur after the cytokine effect fades. Additionally, FCoV-infected cats without FIP were shown to exhibit B and T cell hyperplasia [66,69,70] and increased numbers of circulating T cells [63]. It would thus be of particular interest to further characterize these immunologic features and the cellular immune response against FCoV before and after treatment.

Eosinophilia was noted as another effect of the Xraphconn^®^ treatment in this study. Parasitic infestation was ruled out in most cats, and there was no obvious reason for the eosinophilia. Eosinophil counts were not specifically evaluated in previous studies [24,25,28]. Interestingly, an increase in the eosinophil count has also been found in human patients recovering from COVID-19 and has been proposed as a marker for a favorable outcome [71,72,73]. Whether there was a hypersensitivity reaction resulting in eosinophilia due to treatment in the current study needs to be clarified in further research.

One cat developed mild Heinz body anemia on the last day of therapy. Heinz body formation occurs as a result of oxidative damage to hemoglobin and can result in hemolysis [74]. In cats, increased Heinz body formation has previously been shown to be associated with the administration of certain drugs and components, such as propofol [75], acetaminophen [76], or propylene glycol [77]. Therefore, it seems possible that there is an association between the repeated administration of Xraphconn^®^ and Heinz body formation in this cat. Further studies should include the quantification of Heinz body formation during antiviral treatment in order to clarify a possible causal relationship.

An increase of liver enzyme activity was noted in some cats, but was primarily of a mild to moderate scale. Since GS-441524 is metabolized in the liver, this metabolism could possibly lead to an increased metabolic rate with increased liver enzyme activity. Increases in liver enzyme activity have been reported as adverse effects with this multi-component compound treatment before in an unpublished observation [78]. In the present study, renal toxicity did not occur, which is in agreement with one previously published study [25]. Symmetric dimethylarginine values, considered to be a very sensitive parameter for kidney function, remained within the reference range throughout the treatment. Only one cat with thoracal and abdominal effusion had an SDMA of 18 µmol/L (reference range 0–18 µmol/L) on day 0 before treatment, likely due to impaired perfusion of the kidneys secondary to FIP, but values normalized after treatment was started. Another single cat had renal azotemia and increased SDMA values, which were already present at inclusion into the study. Therefore, it was reasonable to suspect that renal changes before entering the study were caused by FIP. However, in the further course of the study, the cat developed unilateral renal mineralization and mild pyelectasia, which progressed to ureteral obstruction, most likely caused by a ureterolith, after the end of treatment. The cat did not show overt clinical signs related to kidney disease except for the development of a very small amount of ascites. Given that azotemia was already present before the start of Xraphconn^®^ treatment, it seems unlikely that the renal changes represent adverse effects, but rather were the consequence of progressive kidney disease unrelated to the Xraphconn^®^ treatment. In a previous study [25], one cat treated with GS-441524 showed a progressive increase in urea and sudden rise in SDMA concentration during repeated rounds of treatment and as a precaution, it was decided to stop treatment. However, urine specific gravity measurement was not reported in that study, so the presence of renal azotemia, as compared to prerenal azotemia, could not be confirmed. Additionally, urea and SDMA concentrations returned to normal after discontinuation of treatment [25]. In a case report of a cat with FIP treated with the same multi-component compound as in the current study, an increased SDMA was noted during treatment, which normalized after drug discontinuation, although it was not confirmed whether this was as a consequence of antiviral treatment [26].

The question arises as to whether remdesivir, which is licensed for the treatment of humans with severe COVID-19, could be useful for treatment of FIP in cats, Remdesivir has been used by veterinarians in Australia to treat cats with FIP, although published studies are not yet available. GS-44152 is the main metabolite of remdesivir, a prodrug of the nucleoside analogue, which has been used with limited success in patients with acute COVID-19 [29,79]. Remdesivir needs to be applied by injection since it has no acceptable oral bioavailability. In contrast, oral admission of GS-441524 was shown to be effective in mice against COVID-19 [29,80]. Another advantage of GS-441524 over remdesivir appears to be its reduced liver toxicity allowing dose escalation to ensure effective treatment of systemic coronavirus infections [81]; GS-441524 is directly converted into the active nucleoside triphosphate in liver and lung tissue.

The results of the present study can also be viewed from a broader perspective. In the context of the COVID-19 pandemic, pediatricians worldwide have been confronted with a new disease in children, a syndrome associated with SARS-CoV-2 infection and referred to as multisystem inflammatory syndrome in children (MIS-C). When comparing clinical features of FIP with MIS-C, remarkable parallels can be found [5,6]. MIS-C, like FIP, is a hyperinflammatory immune response, primarily seen in the children [5,6]. Affected children initially show often only gastrointestinal signs, while in the course of the disease, further symptoms appear, such as persistent fever, ascites, pleural, and pericardial effusion [5,82,83], all of which are also commonly found in cats with FIP. Therefore, FIP might provide a useful natural model for insights into the pathogenesis and immunology of MIS-C, but also into potential treatment options pending further studies [84]. Finally, studying FIP in cats and the host response to treatment could serve as an interesting natural model for further elucidating features of MIS-C in children. The model could provide critical insights into pathophysiology and immunology of these similar clinical entities and might also help to explore new potential treatment options for this severe SARS-CoV-2-associated disease.

The major limitation of this study was that no untreated control group was included. However, leaving cats with FIP untreated is not justifiable for ethical reasons, and the short median survival time in cats with FIP of eight to nine days clearly has been demonstrated before [9,20]. Thus, without treatment, almost all cats would die within a very short time frame. Another limitation was that the potential efficacy of the additional components in the tablets (other than GS-441524) was not determined in the present study, and measuring any antiviral efficacy of the other components, once successfully identified and purified, would be an interesting additional approach in the future. Due to the oral application of the treatment, cats in a moribund condition could not be included in the study. The two cats that were excluded from the study were in a severely moribund end-stage condition. An oral drug would likely not have had any effect in such a condition, due to the low metabolic rate and the fact that oral medications are hardly absorbed from a likely inactive intestinal tract. Therefore, the cats were not included in the study and instead euthanized for humane reasons. Also, the small sample size of the current study is an additional limitation.

## 5. Conclusions

In conclusion, this study is the first prospective treatment trial clearly demonstrating that cats suffering from this tragic and fatal disease can be cured with oral treatment. Unfortunately, the drug is not currently legally available for veterinary use in many countries, forcing well-meaning owners to self-diagnose and treat their cats based on judgment of non-veterinary lay people and social media groups. Thus, there is an urgent need for respective official bodies and industry to work towards a swift licensing process of the drug so that it can be legally used by veterinary experts to offer supervised treatment to cats suffering from FIP.

## Figures and Tables

**Table 1:** Cats participating in the study, including signalment, number of additional cats in the household, method of diagnosis of feline infectious peritonitis (FIP), FIP-associated signs, Xraphconn^®^ treatment dose, other diseases at the start of and developing during treatment, adverse effects, and additional symptomatic therapy.

Cat	Age (Months)	Sex	Breed	Additional Cats in the Household (Number)	Diagnosis of FIP ^1^	FIP-Associated Cardinal Signs	Dosage (mg/kg q24h)	Adverse Effects and Duration (on Days of Treatment)	Other Unrelated Diseases before Treatment	Other Unrelated Diseases Developing during Treatment	Additional Symptomatic Therapy
cat 1	6.0	male neutered	ESH	yes (1)	immunohisto-chemistry (eye)	ocular signs	10 mg/kg	lymphocytosis (2–end^2^)			fluid therapy ^3^, metamizole ^4^
cat 2	6.3	male intact	ESH	yes (1)	immunohisto-chemistry (eye)	neurologic signs, ocular signs	10 mg/kg	lymphocytosis (0–end)	surgical wound infection after eye enucleation		fluid therapy, antibiotics ^5^, buprenorphine ^6^
cat 3	9.8	male neutered	ESH	yes (1)	RT-PCR detecting S gene mutations (effusion)	abdominal effusion	5 mg/kg	increased liver enzyme activity (4–14)		renal mineraliza-tion	fluid therapy, antibiotics, maropitant ^7^, mirtazapine ^8^
cat 4	7.2	male intact	ESH	yes (1)	RT-PCR detecting S gene mutations (effusion)	abdominal effusion	5 mg/kg	lymphocytosis (2–end), eosinophilia (14–56)			fluid therapy, metamizole-sodium, antibiotics, maropitant, mirtazapine
cat 5	6.4	female intact	ESH	yes (1)	RT-PCR detecting S gene mutations (effusion)	abdominal effusion	5 mg/kg	lymphocytosis (0–end), increased liver enzyme activity (4–83), eosinophilia (14–end)			fluid therapy, antibiotics
cat 6	10.7	male neutered	ESH	yes (1)	RT-PCR detecting S gene mutations (effusion)	abdominal effusion	5 mg/kg	lymphocytosis (83–end)			antibiotics, mirtazapine
cat 7	4.7	male intact	Siamese	yes (1)	RT-PCR detecting S gene mutations (effusion)	abdominal effusion, thoracic effusion	5 mg/kg	lymphocytosis (0–end), increased liver enzyme activity (4–83), eosinophilia (14–end)	chronic gingivo-stomatitis		fluid therapy, antibiotics, silymarin ^9^
cat 8	6.4	male intact	Maine Coon	no	RT-PCR detecting S gene mutations (effusion)	thoracic effusion	5 mg/kg	lymphocytosis (0–14), eosinophilia (14–83)	chronic gingivo- stomatitis		fluid therapy, antibiotics, oxygen cage ^10^
cat 9	8.9	male neutered	ESH	yes (3)	RT-PCR detecting S gene mutations (effusion)	abdominal effusion	5 mg/kg	lymphocytosis (28–end), increased liver enzyme activity (4–83), eosinophilia (28–end)			fluid therapy, antibiotics, mirtazapine, silymarin
cat 10	39.1	female neutered	ESH	yes (3)	immunohisto-chemistry (lymph node)	abdominal effusion	5 mg/kg	eosinophilia (0–end)	intestinal parasite infestation (*Giardia* spp., treated with fenbendazole)		
cat 11	56.5	female neutered	ESH	yes (3)	RT-PCR detecting S gene mutations (effusion)	abdominal effusion	5 mg/kg	lymphocytosis (0–14), increased liver enzyme activity (4–97)			fluid therapy, antibiotics
cat 12	11.7	male neutered	Birman	yes (1)	immunohisto-chemistry (lymphnode), RT-PCR detecting S gene mutations (effusion)	thoracic effusion	5 mg/kg	lymphocytosis (2–end), increased liver enzyme activity (28–56), eosinophilia (28–83)			fluid therapy, antibiotics, buprenorphine
cat 13	28.8	female intact	Maine Coon	yes (9)	RT-PCR detecting S gene mutations (effusion)	thoracic effusion	5 mg/kg	eosinophilia (28–end)	rhinitis		fluid therapy, antibiotics
cat 14	7.5	male intact	ESH	yes (1)	RT-PCR detecting S gene mutations (effusion)	abdominal effusion	5 mg/kg			intestinal parasite infestation (*Giardia* spp., treated with fenbendazole)	fluid therapy, antibiotics, maropitant, mirtazapine, buprenorphine, pregabalin ^11^
cat 15	7.6	male intact	Maine Coon	yes (1)	RT-PCR detecting S gene mutations (effusion)	abdominal effusion	5 mg/kg	lymphocytosis (2–56), eosinophilia (2–28)		distorsion on right forelimb	fluid therapy, antibiotics, buprenorphine, meloxicam ^12^
cat 16	8.9	female neutered	BSH	yes (1)	RT-PCR detecting S gene mutations (effusion)	abdominal effusion	5 mg/kg	lymphocytosis (7–14), increased liver enzyme activity (2–14)	chronic gingivo-stomatitis		fluid therapy, mirtazapine
cat 17	7.7	male intact	Scottish Fold	yes (1)	RT-PCR detecting S gene mutations (effusion)	thoracic effusion	5 mg/kg	lymphocytosis (2–end), eosinophilia (28–end)	otitis externa	pyothorax	fluid therapy, antibiotics
cat 18	7.6	female intact	ESH	yes (2)	RT-PCR detecting S gene mutations (effusion)	abdominal effusion	5 mg/kg	lymphocytosis (0–end), eosinophilia (2–end)			fluid therapy, antibiotics

mg, milligram; kg, kilogram; q24h, every 24 h; ESH, European shorthair; RT-PCR, reverse transcription polymerase chain reaction; S, spike; BSH, British shorthair; spp., species. ^1^ diagnosis of FIP: FIP confirmed: positive IHC plus consistent histopathology; FIP very likely: positive RT-PCR and analysis for FCoV spike mutation. ^2^ end: until the end of the observation period. ^3^ fluid therapy with Ringer’s lactate with potassium supplementation at 20 mval/L for dehydration at an individual dosage calculated by rehydration and maintenance needs. ^4^ metamizole 30 mg/kg intravenously (IV) for treatment of fever (body temperature > 40.5 °C) as a single injection. ^5^ antibiotics (i.e., amoxicillin/clavulanic acid 20 mg/kg q8h IV or per os (PO); trimethoprim sulfadiazin 20 mg/kg q12h IV or PO; marbofloxacin 2 mg/kg q24h IV; pradofloxacin 6 mg/kg q24h PO; ampicillin 12.5 mg/kg q8h IV for treatment of secondary suspected or proven bacterial infection (neutrophilia with left shift or continuously high body temperature). ^6^ buprenorphine 0.01 mg/kg q8h IV for treatment of pain. ^7^ maropitant 1 mg/kg q24h IV for treatment of gastrointestinal signs, such as vomiting and anorexia. ^8^ mirtazapine ointment q24h for appetite stimulation. ^9^ silymarin 20 mg/kg q12h PO for 10 days and then 20 mg/kg q24h PO for treatment of increased liver enzymes. ^10^ oxygen cage for support in cases of dyspnea in cats with massive thoracic effusion. ^11^ pregabalin 2 mg/kg q12h PO when buprenorphine was not sufficient to control pain. ^12^ meloxicam 0.1 mg/kg q24h PO for 1 day and then 0.05 mg/kg q24h PO for treatment of orthopedic pain.

**Table 2:** Adverse effects of the Xraphconn^®^ treatment, grades of adverse effects, day of first appearance, and respective symptomatic treatment. ^1^ Heinz body formation: mild (5–9.9% of red blood cells), moderate (10–25% of red blood cells), severe (>25% of red blood cells); ^2^ lymphocytosis: mild (4–7.9 × 10^9^/L), moderate (8–15 × 10^9^/L), severe (>15 × 10^9^/L) ^3^ was already present on day 0: lymphocytosis: 5/14 cats; eosinophilia: 1/11 ^4^ eosinophilia: mild (0.6–1.9 × 10^9^/L), moderate (2–10 × 10^9^/L), severe (>10 × 10^9^/L); ^5^ increased liver enzymes: increased alanine aminotransferase (ALT) activity: mild (ALT < 200 IU/L), moderate (ALT 200–350 IU/L), severe (ALT >350 IU/L); increased alkaline phosphatase (AP): mild (AP < 200 IU/L), moderate (AP 200–350 IU/L), severe (AP > 350 IU/L).

Adverse Effect		Number of Cats	Grade	Median Day of First Appearance (Range)	Symptomatic Treatment
Heinz body formation ^1^		1/18	moderate	83	S-adenosyl-methionine
	14/18 ^3^	4/14	mild	4.5 (2–83)	no
Lymphocytosis ^2^		6/14	moderate	1 (0–28)	no
		4/14	severe	1 (0–2)	no
Eosinophilia ^4^	11/18 ^3^	11/11	mild	14 (0–28)	no
increased liver enzyme activity ^5^		8/11	mild	28 (2–83)	no
	11/18	1/11	moderate	4	no
		2/11	severe	4	silymarin

## Data Availability

The authors confirm that the datasets analyzed during the study are available from the corresponding author upon reasonable request.

## Supplementary Materials

The following are available online at https://www.mdpi.com/1999-4915/13/11/2228/s1, File S1: Package inserts of Xraphconn^®^. (A) Package insert of first batch. (B) Package insert of second batch. Table S1: Schedule of examinations performed in all cats (days on which examinations took place are marked in grey). FCoV, feline coronavirus; ^1^ ultrasound and paracentesis: if abdominal or thoracic effusion was present, additional ultrasounds of abdomen (and/or thorax) were performed daily from day 0 to day 7 to evaluate fluid amount and obtain fluid samples; as long as effusion was present, effusion viral load was determined in addition. ^2^ including symmetric dimethylarginine (SDMA) and serum amyloid A (SAA). able S2: Karnofsky’s score modified for cats by Hartmann and Kuffer (1998) to evaluate the general condition and well-being [40]. Text S1: Characterization of the active ingredient in Xraphconn^®^. Table S3: 1H and 13C chemical shifts in ppm for the nucleoside analogue extracted from Xraphconn^®^ compared to data reported on pure GS-441524 in fully deuterated dimethyl sulfoxide (DMSO-d6). nr, not reported; splitting pattern of proton signals (s, singlet; d, doublet; t, triplet; q, quartet; dd, double doublet; app q, apparent quartet). Figure S1: Percentage of cats with effusion during study course. The bars represent percentage of cats with effusion on each examination day. The grey areas represent the proportion of cats in which effusion was present but not available for paracentesis. The green areas represent the proportion of cats in which effusion could be obtained but was feline coronavirus (FCoV) RNA-negative. The orange areas represent the proportion of cats in which effusion could be obtained and was RNA-positive. Figure S2: Anti-feline coronavirus (FCoV) antibody titers throughout the study course. Figure showing predictive values and 95% confidence intervals of antibody titer. Samples were tested at dilutions of 1:25, 1:100, 1:400, 1:1600 and 1:6400. Red asterisks mark significant difference (*p* ≤ 0.05) of the parameters on different days of treatment when compared to day 0 (before treatment) measured by a robust linear mixed-effect model.
