# Supplementary material for: Curing Cats with Feline Infectious Peritonitis with an Oral Multi-Component Drug Containing GS-441524
Source: Viruses. 2021 Nov 5;13(11):2228. doi: 10.3390/v13112228 (PMC8621566; doi:10.3390/v13112228)
Supplement: Supplementary file 1 [file viruses-13-02228-s001.zip › File S1.pdf]

A

MUTIAN®
www.mutian.com

Shelf Life: Printed on the package

Xraphconn®
tablets

For use in cats only.
Not for use in humans.

[Description]
Xraphconn® is a compound tablet that combats the feline infectious peritonitis virus. It contains 200 mg of Radix scrophulariae, Platycodon grandiflorum, Phyllostachys pubescens, Forsythia suspensa, Anemarrhena asphodeloides, and MT0901, and other active ingredients. These are inactive ingredients: Microcrystalline Cellulose, Polyvinylpolypyrrolidone cross-linked, Magnesium stearate.

The structural formula of MT0901 is:

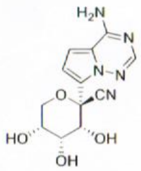

Molecular Formula: $C_{12}H_{13}N_5O_4$ Molecular Weight: 291.27

[Product Properties]
It occurs as a pale yellow film-coated tablet.

[Product Specifications]
200 mg*20s

[Indication]
Xraphconn® tablets are used to treat cats suffering from various forms of feline infectious peritonitis (FIP).

[Dosage and Administration]
Oral: Do not administer antacids, sucralfate, or multivitamins/supplements containing iron, zinc, magnesium, or aluminum for at least 2 hours before or after giving Xraphconn®. Administer on an empty stomach with water (at least 1 hour before or 2 hours after meals, milk, or other dairy products). **Consult your veterinarian for help in the diagnosis, treatment and control of FIP.**

- **Choose the dosage based on the cat's body weight: The daily dose for Xraphconn® is 100 mg/kg of body weight.**
- **Dosage adjustment: Symptoms of FIP, disease progress and denouement are varied due to the age and health condition of the cats. The veterinarian should assess the clinical condition of the cat carefully. 1.5 times the dosage is recommended if uveitis or ocular symptoms are present. 2 times the dosage is recommended if neurosis occurs.**
- **Duration of treatment: 12 weeks is recommended as a course of treatment.**

[Contraindications]
It is contraindicated in cats with a known hypersensitivity to any ingredient of Xraphconn®.

[Precautions]
Xraphconn is not recommended in cats with severe hepatic impairment and/or severe renal impairment. Safety and efficacy data were not available for kittens under 6 months.

[Human warnings]
For cat use only. Not for use in humans. Keep this and all medications out of reach of children and pets. Consult a physician in case of accidental ingestion by humans.

[Adverse Reactions]
In a randomized, double-blind, vehicle-controlled field study to assess the effectiveness and safety of xraphconn for FIP, 134 cats treated with xraphconn and 19 cats treated with vehicle control were evaluated for safety. The vehicle control was a tablet with the same appearance as the xraphconn but without the active ingredient. During the 12-week trial, the most common adverse reactions included immediate reactions, diarrhea or soft stool, and behavioral abnormalities (vocalization, hyperactivity, and attention seeking dabbed). No serious AEs occurred in patients administered xraphconn. No AEs led to discontinuation. There were no opportunistic infections, malignancies, gastrointestinal perforations, serious kidney and/or liver damage, adjudicated cardiovascular events, or thromboembolic events. No grade 3 or 4 clinical events were considered to be related to the drug by the investigators, and all deaths were associated with uncontrolled disease progression. The serum phosphorus (P) increased in 16 cats, the serum urea nitrogen (BUN) increased in 7 cats, and the serum creatinine (SCr) increased in 2 cats during the study. All renal dysfunction AEs were considered mild, and the increases in BUN and P were considered to be related to a high-protein and/or high-phosphorus diet and were resolved without any change in dosage. One cat with renal insufficiency was reported with a serious adverse reaction of acute renal failure, hematuria, and pyuria during the Week 7 visit. The cat was enrolled with a history of chronic kidney disease. Euthanasia was elected, and the necropsy revealed hypertrophic cardiomyopathy, bilateral parathyroid hyperplasia, and mild to moderate renal disease. Alanine aminotransferase (ALT) increased in 13 cats, and creatine phosphorkinase (CK) increased in 6 cats (neither event was grade ≥ 2 laboratory increase). All AEs of ALT and CK elevation were asymptomatic in cats receiving xraphconn and reported as mild in severity. The adverse reactions observed in the study and the number of cats experiencing each adverse reaction are summarized in Table 1 below. The vehicle-controlled group was not involved in the statistical analysis because no cats were still alive at week 12.

Table 1. Adverse Reactions Reported During the Field Study

Adverse Reaction	Xraphconn® n=134 (%)
Behavioral	
Immediate Reaction *	89 (66%)

Continued on Next Page

Adverse Reaction	Xraphconn® n=134 (%)
Behavioral	
Immediate Reaction *	89 (66%)
Vocalization	23 (17%)
Hyperactivity	17 (13%)
Attention Seeking	13 (10%)
Aggression	4 (3%)
Physical Examination or Observational	
Diarrhea or Soft Stool	16 (12%)
Frequent Vomiting	7 (5%)
Polydipsia and polyuria	5 (4%)
Obesity	4 (3%)
Flu-like Symptoms	4 (3%)
Constipation	2 (1%)
Clinical Pathology	
Elevated P	16 (12%)
Elevated ALT	13 (10%)
Elevated BUN	7 (5%)
Elevated CK	6 (4%)
Elevated Crea	2 (1%)
Pancreatitis	2 (1%)
Urinary tract infection	1 (1%)
Death	8 (6%)

* Cats often experience immediate reactions during xraphconn treatment, including salivation, nausea, vomiting, irritability, etc.

P, phosphorus, ALT, alanine aminotransferase. BUN, blood urea nitrogen. CK, creatine phosphokinase. Crea, creatinine.

[Clinical Pharmacokinetics]
The drug plasma concentration changes in healthy specific pathogen free (SPF) cats of 6-9 months (n = 12) following a single dose of 100 mg/kg xraphconn administered orally. Serial blood samples were then collected, and the plasma drug concentration of MT0901 (the active ingredient of xraphconn) was measured. MT0901 is an inhibitor of FIPV RNA-dependent RNA polymerase (RdRp), which is essential for viral replication. MT0901 is a nucleoside monophosphate that distributes into cells. The nucleoside monophosphate is subsequently phosphorylated by cellular kinases to form the pharmacologically active nucleoside triphosphate metabolite. MT0901 triphosphate (MT0901-TP) acts as an analog of adenosine triphosphate (ATP) and competes with high selectivity (3.83-fold) over natural ATP substrates for incorporation into nascent RNA chains by the FIPV RNA-dependent RNA polymerase, which results in delayed chain termination (position i+3) during viral RNA replication. The pharmacokinetic (PK) properties of MT0901 is provided in Table 2. The multiple dose PK parameters of MT0901 is provided in Table 3.

Table 2. Pharmacokinetic Properties of Xraphconn.

**Absorption**	
T_{max} (h)*	1.51–2.00
**Distribution**	
% bound to human plasma proteins	88–93.6†
Blood-to-plasma ratio	0.68–1.0
**Elimination**	
$T_{1/2}$ (h)‡	3.56-5.21
**Metabolism**	
Metabolic pathway(s)	CES1 (80%)<br>Cathepsin A (10%)<br>CYP3A (10%)
**Excretion**	
Major route of elimination	Metabolism
% of dose excreted in urine§	46
% of dose excreted in feces§	ND

* After oral administration of MT0901 for 30 minutes, range of median observed on Day 1 and Day 5 or 10. † Range of protein binding for MT0901 from 2 independent experiments show no evidence of concentration-dependent protein binding for MT0901.
‡ Median. § Mean. ND=not detected

Table 2. Multiple Dose PK Parameters* of Xraphconn Following PO Administration of Xraphconn Tablets 100 mg/kg/dose to Healthy Cats

Parameter Mean (CV%)	
C_{max} (nanogram per mL)	2229 (19.2)
AUC_{tau} (nanogram·h per mL)	1585 (16.6)
C_{trough} (nanogram per mL)	ND

CV=Coefficient of Variation; ND=Not detectable (at 24 hours post-dose)
* After oral administration of MT0901 for 30 minutes

[Effectiveness]
The effectiveness of xraphconn was demonstrated in a randomized, double-blind, vehicle-controlled, multi-site field study involving client-owned cats of various breeds. The inclusion criterion for entering the study was the verified diagnosis of FIP. Cats were randomly administered xraphconn (100 mg/kg, PO, q24h) or the same volume of placebo for 12 weeks. When the condition worsened and to alleviate suffering, veterinarians were allowed to select any approved drugs to administer if needed. The primary endpoint was overall survival (OS). The secondary end points were clinical complete remission (CR) rate and safety; additional end points included proportions of cats achieving a Karnofsky's score of 100 at each visit. Between Feb 3, 2019, and Aug 17, 2019, 219 cats were screened, of whom 154 were eligible. 134 cats were assigned to receive xraphconn and 20 to receive placebo; a cat owner in the placebo group withdrew their previously written informed consent after randomization, so 134 and 19 cats were included in this trial. There was a large imbalance between the number of cats in the xraphconn group and those in the placebo group because historical data on untreated cats was available, demonstrating a mortality rate close to 100%[1]. The median age of the cats in the study was 10.5 months. Sex distribution was 79 (59%) males versus 55 (41%) females in the xraphconn group and 13 (68%) versus 6 (32%) in the placebo group. There were significant differences in OS, CR, and all other variables at every stage after starting treatment between the xraphconn group and the placebo group. The xraphconn group produced 126 (94%) long-term survivors, however, cats in the placebo group survived only 2-55 days (median, 9.5 days). All deaths were associated with uncontrolled disease progression. Xraphconn efficacy was generally demonstrated by day 1 to 3, with peak values have been reached and maintained after week 4 to 6. Of the 134 cats treated by xraphconn, 117 cats were observed as reaching the asymptomatic period within two weeks of initial treatment, and 126 cats achieved complete clinical remission at week 12. However, 2 (1.6%) cats relapsed (day 3, 11, respectively) after 12 weeks of treatment These 2 cats were successfully treated at a second round a[illegible] the higher dosage (200 mg/kg, PO, q24h). Final follow-up[illegible] were on April 10, 2020, more than three months after th[illegible] last cat stopped taking xraphconn (January 7, 2020) and 12[illegible] cats remain alive and well. The results showed that xraphconn seems to be a potential game changer, compared with placebo, xraphconn showed an overwhelming efficacy.

[Four-week safety study of xraphconn in cats]
After the dosage regimen of xraphconn was determined in the PK study, safety of xraphconn was evaluated in 12 healthy SPF cats of 6–9 months of age. The cats were administered with 100 mg/kg xraphconn tablets once daily at 10 AM for [illegible] weeks. For the duration of the study, they were observed daily for adverse effects. Blood samples were taken weekly and the complete blood counts and blood chemistry panels were conducted. During the study period, there were no clinically significant changes in vital signs and clinical lab parameters, indicating that the dosage and the route o[illegible] administration of xraphconn was well-tolerated in cats fo[illegible] the duration of the safety study.

[Storage Conditions]
Store below 30°C (86°F)

[Expiration date]
24 months

REFERENCES

[1] Pedersen NC, Eckstrand C, Liu H, Leutenegger C, Murphy B. Levels of feline infectious peritonitis virus in blood, effusions, and various tissues and the role of lymphopenia in disease outcomes following experimental infection. Vet Microbiol. 2015; 175(2-4):157-66.

PATENT PENDING
MT0901 is a patented compound of Mutian Life Sciences Co., Ltd.

Manufactured under the oversight of a Quality Managemen[illegible] System, which meets the requirements of ISO 9001:2000, IS[illegible] 22716:2007, CGMP

MUTIAN
木天生物

Manufactured for: Mutian Life Sciences Ltd
98# Jianghai Industry Zone, Tongzhou, Nantong, Jiangsu, China.
www.mutian.com

Xraphconn is a trademark of Mutian Life Sciences Co., Ltd.
©2020 Mutian Life Sciences Co., Ltd.

All rights reserved.

B

Storage	Prevent moisture and store airtight.

OTC Animal Drugs

MUTIAN®
xraphconn

Shelf Life: Printed on the package

[Main Ingredient]
Radix scrophulariae, Platycodon grandiflorum, Phyllostachys pubescens, Forsythia suspensa, Anemarrhena asphodeloides, etc.

[Ingredients of the coating]
Hydroxypropyl cellulose, PEG 6000, Crystalline cellulose, Titanium oxide.

[Product Properties]
It occurs as a pale yellow film-coated tablet.

[Indications]
Xraphconn tablets are used to treat cats suffering from various forms of feline infectious peritonitis (FIP).

[Product Specifications]
200mg

[Recommended Dosage]
Consult your veterinarian for help in the diagnosis, treatment and control of FIP.

- Choose the dosage according to the cat's body weight: The daily dose for Xraphconn is 100 mg/kg of body weight. It is recommended to take Xraphconn tablets on an empty stomach. If vomiting occurs after 30 mins from dosage, another dose is not needed.
- Dosage adjustment: Symptoms of FIP, course of disease and denouement are varied due to the age, health condition of the cats. The veterinarian should assess the clinical condition of the cat carefully. 1.5 times of dosage is recommended if uveitis or ocular symptoms occurs. 2 times of dosage is recommended if neurosis occurs.
- Duration of treatment: 12 weeks is recommended as a course of treatment.

[Adverse effects]
No obvious adverse effects were seen during the clinical trials. In clinical studies 7 of 240 cats (about 3% of the treated cats) had vomiting associated with the use of the product.

[Contraindications]
Safe use in pregnant animals or animals intended for breeding has not been proven. If animal' s condition worsens or does not improve, stop product administration and consult your veterinarian. **For use in cats only.**

[Precautions]

1. Prevent medicine withdrawal which may increase drug resistance. Monitor the cat's body weight and adjust the dosage accordingly with weight gain.
2. Cats with renal insufficiency: In clinical trials, some cats with serum SDMA and/or CREA level increased were observed, but this may be affected by age, food, basic diseases and other factors. We can't exclude the increasing possibility of serum SDMA and /or CREA level during the treatment of infectious peritonitis with Xraphconn. Therefore, it is suggested to monitor the renal indexes before and during the treatment. If CREA increases by more than 1.8 mg/dl, suspension of treatment or dose reduction may be considered.
3. Cats with severe liver dysfunction: The efficacy and safety of Xraphconn for cats with severe liver dysfunction are not clear yet. The risk / benefit of the cat should be carefully assessed as well as the liver function of the cat should be closely monitored during the treatment.

[Drug Interaction]
The combination of Xraphconn and Compound Aluminium Hydroxide or Omeprazole may lead to plasma Xraphconn area under curve (AUC) by 49% and 56%, while the plasma concentration (C_{max}) decreases by 46% and 52% respectively. Please avoid alkaline drugs and proton pump inhibitors while using Xraphconn tablets.

[Pharmacokinetics]
The dose of Xraphconn for cats is 100 mg/kg. The peak concentration (C_{max}) is 5.48±1.10 μg/ml, the peak time (T_{max}) is 2.00±1.10h, the half-life time ($T_{1/2}$) is 4.86 ± 0.72h, the area under curve (AUC_{0-t}) is 37.88 ± 3.91 ng/ml *h.

[Clinical trials]

A total of 186 cats with FIP were included in the study called "How Xraphconn affect Feline Infectious Peritonitis (FIP) living quality and time". Veterinarians from Britain, the United States, Italy, Germany, Japan, France, Romania, Turkey and China participated in this project. Due to ethics, there was no placebo control group. The results are as follows:

- 5 of 186 cats died or were euthanized within 6 days of the initial treatment.
- 10 of 181 cats recovered after 4-8 weeks of treatment, and the remaining 171 cats recovered after 12 weeks of treatment.
- 13 of the 181 cats were those who had relapsed after already having had treatments with nucleoside analogs where that treatment was unsuccessful. After beginning treatment with *Xraphconn* (100-400 mg/kg p.o. q24h), 11 of the 13 recovered, including 2 cats with ocular lesions and 5 cats with neurological symptoms. A further two cats still relapsed after completing 12 weeks of treatment however both of these cats had neurological symptoms before treatment, and are currently receiving a further round of treatment at three or four times the dose. Through blood concentration analysis, it can be found that their tolerance to *Xraphconn* has nothing to do with the rate of drug absorption. The treatment effect is unsatisfactory most likely due to the individual's resistance, and they may require higher doses of the medication for a longer period of time.
- 147 cats have stopped medication from between 1 and 8 months, and have had no recurrence of FIP, with 23 cats still under observation. It should be noted that cats with ocular lesions and neurological symptoms were not included in this investigation. It was found that the possibility of complications increased if the cat with FIP has ocular lesions or neurological symptoms, and there will be an increased chance of relapse after the treatment.
- 2 of 181 cats died two months post treatment. One of the cats suffered a sudden cardiac arrest. In this case, there was an abnormal cardiac fiber, coagulation necrosis, disappearance of the nucleus and striated muscle, and soaking of neutrophilic granulocyte. The second cat suffered from kidney problems (CREA: 3.4 mg/dL, BUN: 49 mg/dL) before the treatment. It is unclear if the death was linked to the administration of *Xraphconn*. No other obvious adverse effects occurred in any of the other cats during the trial.

[Effectiveness]

Clinical evaluation:

The cats' health improved within 1-3 days of taking the Xraphconn tablets, they quickly returned to normal body temperature, activity and appetite. In most cases, ascites and/or pleural effusion in cats with wet FIP disappear within 2-3 weeks after treatment.

Comments from veterinarians and cat owners:

The Xraphconn tablets have been highly praised by veterinarians for their effectiveness as most of the cats have improved their health within 24 hours of taking the tablets. Cat owners gave a good response on KPS which over 90% of the cats got a score of 100 within two weeks.

[Safety Evaluation]

During the 30-day drug safety study, three groups of experimental cats (three in each group) were administered either the conventional dose of 100 mg/kg, or double dose (200 mg/kg), or a quadruple dose (400 mg/kg), and their blood cells, systemic metabolism, blood coagulation, ophthalmology, urine routine, and ECG were examined. The results showed that the drug had no obvious adverse reactions.

[Overdose]

The maximum tolerable dosage for cats has not been established.

[Storage Conditions]

Prevent moisture and store airtight.

[Packaging]

Aluminum strip packaging with 10 tablets in a strip, 20 tablets in a box.

[Expiration date]

24 months

PATENT PENDING

Manufactured under the control of a Quality Management System, which meets the requirement of ISO 9001:2000, ISO 22716:2007, CGMP

- Licensed by the China Ministry of Health

Manufactured for: Mutian Life Sciences Ltd

98# Jianghai Industry Zone, Tongzhou, Nantong, Jiangsu, China.

Xraphconn is a trademark of Mutian Life Sciences Co., Ltd.
© Mutian Life Sciences Co., Ltd. Version: 1.06.
