# Supplementary material for: Curing Cats with Feline Infectious Peritonitis with an Oral Multi-Component Drug Containing GS-441524
Source: Viruses. 2021 Nov 5;13(11):2228. doi: 10.3390/v13112228 (PMC8621566; doi:10.3390/v13112228)
Supplement: Supplementary file 1 [file viruses-13-02228-s001.zip › Table S2.pdf]

0%	dead
10%	severely diseased
20%	major changes in the general condition
30%	medium changes in the general condition
40%	minor changes in the general condition
50%	completely normal general condition
