# Supplementary material for: Curing Cats with Feline Infectious Peritonitis with an Oral Multi-Component Drug Containing GS-441524
Source: Viruses. 2021 Nov 5;13(11):2228. doi: 10.3390/v13112228 (PMC8621566; doi:10.3390/v13112228)
Supplement: Supplementary file 1 [file viruses-13-02228-s001.zip › Table S3.pdf]

	Xraphconn®	GS-441524
H-2'	4.63 (d)	4.94 (d)
H-3'	3.95 (t)	4.35 (t)
H-4'	4.05 (q)	4.42 (app q)
H-5'/H-5"	3.62(dd)/3.48 (dd)	3.86 (dd)/3.79 (dd)
HO-2'	6.15	nr
HO-3'	5.29	nr
HO-5'	5.02	nr
H-2	7.90 (s)	8.1(s)
H-7	6.91(d)	7.37 (d)
H-8	6.88(d)	7.14 (d)

	Xraphconn®	GS-441524
C-1'	78.7	78.6
C-2'	74.6	74.2
C-3'	70.4	70
C-4'	85.8	85.4
C-5'	61.3	60.9
CN	117.8	117.3
C-2	148.3	147.9
C-5	116.8	116.5
C-6	155.9	155.6
C-7	101.4	100.8
C-8	111.2	110.8
