# Supplementary material for: Curing Cats with Feline Infectious Peritonitis with an Oral Multi-Component Drug Containing GS-441524
Source: Viruses. 2021 Nov 5;13(11):2228. doi: 10.3390/v13112228 (PMC8621566; doi:10.3390/v13112228)
Supplement: Supplementary file 1 [file viruses-13-02228-s001.zip › Text S1.pdf]

Ultra-High-Performance-Liquid Chromatography Electro-Spray QTRAP Mass Spectrometry (UHPLC-ESI-QTRAP-MS)*:* All measurements were performed on an Agilent 1290 UHPLC coupled to a QTRAP 6500+ mass spectrometer (MS) with a Turbo-V ESI source from Sciex (Singapore). The UHPLC system comprised of a binary pump, a multi-sampler, and a column oven from Agilent Technologies (Waldbronn, Germany). The MS/MS measurements were performed with a curtain gas of 30 psi, collision gas medium, ion source gas 1 (GS1) 40 psi, GS2 40 psi, declustering potential (DP) 100 eV, entrance potential (EP) 10 eV, collision energy (CE) 50 eV, CXP of 10 eV, ion spray voltage (IS) of 4500 eV and a source temperature of 550°C. The multiple reaction monitoring (MRM) settings were positive ionization mode, Q1 *m/z* 292.104, Q3 *m/z* 163.050, dwell time 20 msec. Information-dependent acquisition (IDA) was performed with *m/z* 292.104 in the inclusion list for fragmentation at 1 minute. The IDA settings were 1 to 2 most intense peaks for ions greater than *m/z* 70 and smaller than *m/z* 1000, which exceeds 500 cps. In addition, former target ions were never excluded and a mass tolerance of 250 mDa was chosen. The enhanced product ion scan (EPI) was performed with 2 experiments and a scan rate of 10,000 Da/s. Liquid chromatography was performed on a CORTECS UHPLC T3 2.1 mm x 50 mm column 1.6 µm particle size connected with the corresponding VanGuard column from Waters GmbH (Eschborn, Germany). All solvents and formic acid were of LC-MS grade and purchased from Sigma Aldrich (Germany). The GS-441524 standard was obtained from ABCR (Karlsruhe, Germany). Mobile phase A (MP-A) was 0.05% formic acid in water and MP-B was 0.05% formic acid in acetonitrile. For this analysis, a very short gradient run with a total run time of 5 minutes was chosen: 0-0.3 minutes with 5% MP-B, 0.3-0-35 minutes with 30%, 0.35-1.50 minutes with 70%, 1.50-1.80 minutes to 90%, 1.80-2.80 minutes with 90%, 2.80-2.90 minutes with 5% and 2.90-4.10 minutes with 5% MP-B for column regeneration. A flow rate of 0.4 ml/minute, a column temperature of 40°C and an injection volume of 1.5 µl were chosen. Concerning the extraction of the drug product for LC-ESI-QTRAP-MS/MS analysis, the Xraphconn® tablet (50 mg drug, 90.5 mg dry weight)

was crushed to a fine powder. The compound was well-emersed in dried plant material, containing most probably polyphenolic compounds such as tannins and lignins. In total, 75.6 mg of the powder were extracted with 756 µl MeOH to which 30 µl 1 M aqueous HCl was added until a pH of 3 was obtained. After vortexing for 2 minutes, the extract was centrifuged for 10 minutes at 13,000 rpm and 4°C. The residue was re-suspended in 0.5 ml MeOH with 10 µl 1 M HCl and was extracted as previously described. 200 µl of the pooled extract was evaporated under flowing nitrogen to 50 µl. Upon addition of 150 µl Aqua dest., a precipitate was formed, which was removed by 20 minutes centrifugation. LC-MS/MS analysis was performed with 1.5 µl of 1:10, 1:100 and 1:1000 dilutions with water. Higher dilution factors were advantageous in obtaining nice MS/MS spectra, since no pre-purification of the extract was performed.

Nuclear magnetic resonance (NMR) spectroscopy: To extract the nucleoside analogue in Xraphconn® for NMR, 3 tablets were molded into a powder that was suspended in 15 ml H_2O. The pH of the suspension was adjusted to 3 by adding 500 µl HCl 1M in H_2O, mildly simulating gastric conditions. At this pH, the nucleobase of the nucleoside analogue is protonated, which increases its solubility under aqueous conditions. After centrifugation (1,100 rpm for 7 minutes), an additional purification step was applied on the collected supernatant: pH was adjusted to 7 by adding 240 µl NaOH 1M in H_2O to induce precipitation of the nucleoside analogue due to deprotonation of the nucleobase. Centrifugation was used to separate the precipitate from the supernatant. After lyophilization, 1 ml Dimethyl sulfoxide-d6 (DMSO-d6) and 50 µl 0.1M HCl in D_2O were added to the precipitate (23 mg) and the saturated solution was analyzed by NMR using a Bruker Avance II 600 MHz at 293 K equipped with 5 mm TCI cryoprobe. The HOD signal was suppressed by applying pre-saturation during a relaxation delay for 1 second in proton detected 1- and 2-dimensional spectra.
