# Supplementary figures and images for: Curing Cats with Feline Infectious Peritonitis with an Oral Multi-Component Drug Containing GS-441524
Source: Viruses. 2021 Nov 5;13(11):2228. doi: 10.3390/v13112228 (PMC8621566; doi:10.3390/v13112228)

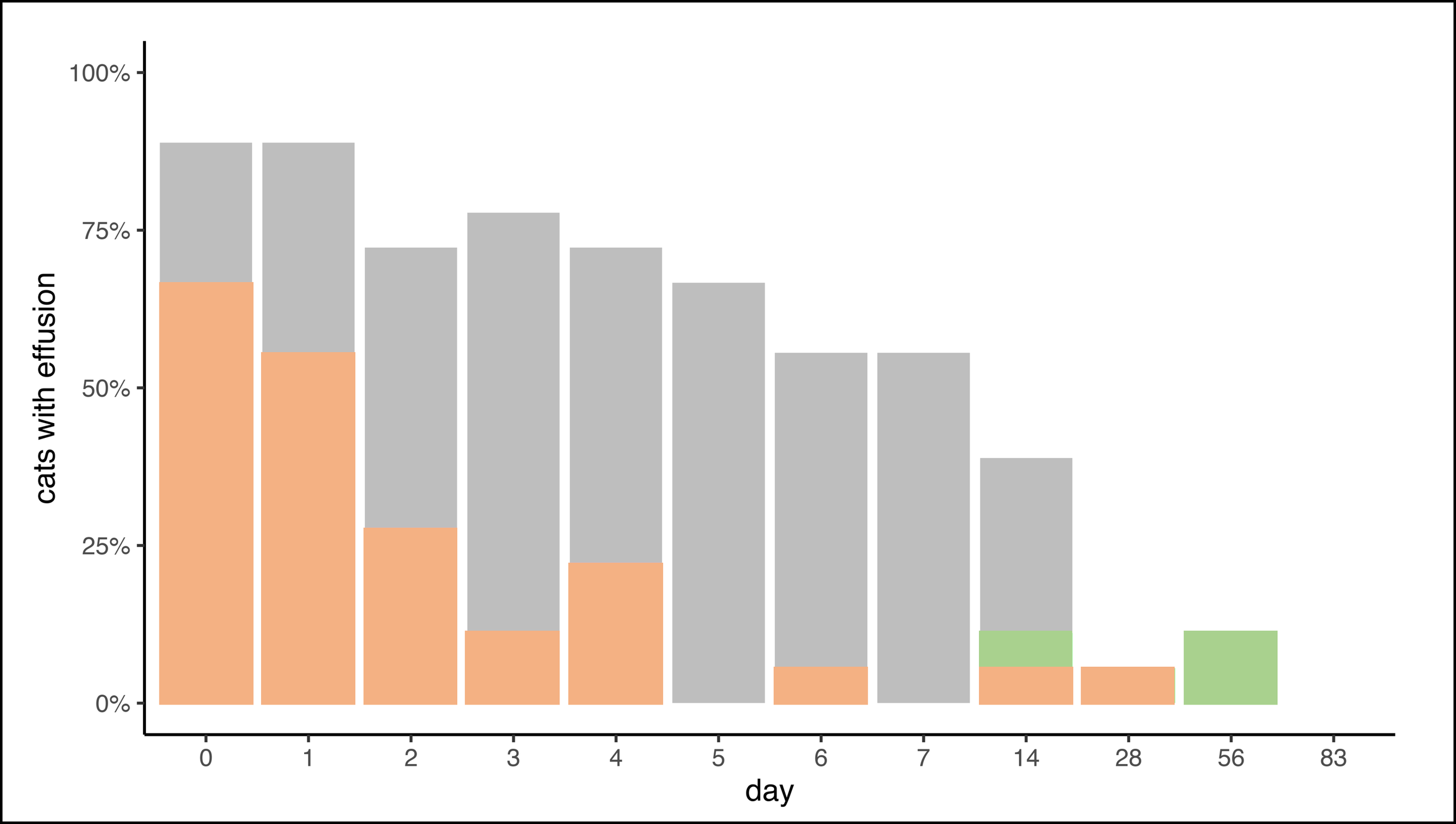

Supplement: Supplementary file 1 [file viruses-13-02228-s001.zip › Figure S1.tif]

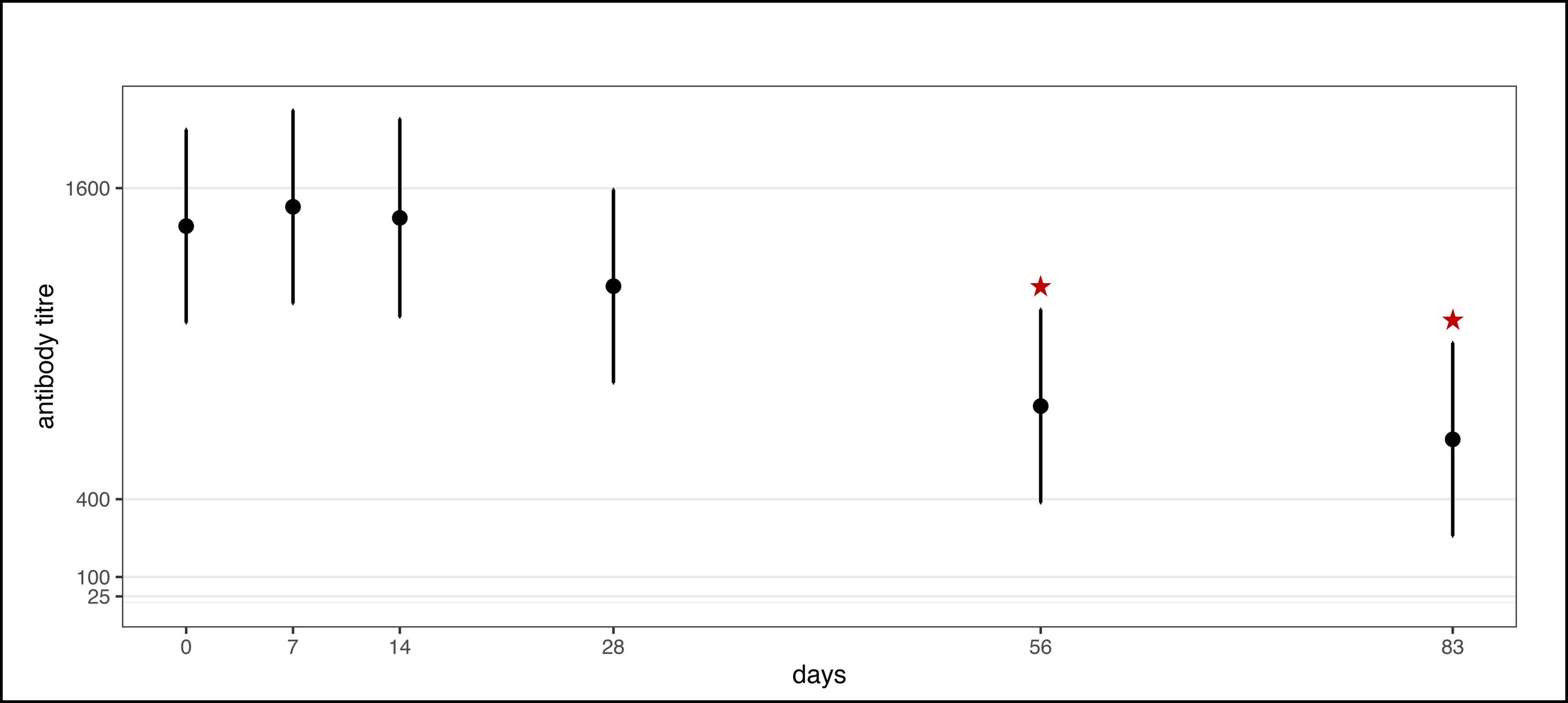

Supplement: Supplementary file 1 [file viruses-13-02228-s001.zip › Figure S2.jpg]
